# Supplementary material for: Understanding data and information needs for palliative cancer care to inform digital health intervention development in Nigeria, Uganda and Zimbabwe: protocol for a multicountry qualitative study
Source: BMJ Open. 2019 Oct 31;9(10):e032166. doi: 10.1136/bmjopen-2019-032166 (PMC6830840; doi:10.1136/bmjopen-2019-032166)
Supplement: Supplementary data [file bmjopen-2019-032166supp001.pdf]

Appendix A: Palliative care reports undertaken in participating countries over the last 15 years

Country	Report	Key findings
Nigeria	Federal Ministry of Health: Treat the Pain (2012 – 2015):	• Around 177,000 people were estimated to have died in moderate or severe pain from HIV or cancer.<br>• The utilized narcotic medicines such as morphine was enough to treat only 266 people, representing 0.2% coverage of pain treatment
	Hospital Pain Free Initiative (2015 – date):	• Program to improve access to essential pain medicines to reduce needless suffering from cancer<br>• Pharmacist employed into Federal Ministry of Health specifically to coordinate the project<br>• 19.2kg of pulverized morphine imported, enough to treat about 3,000 patients.<br>• Strengthening the skills of health workers in pain assessment and management and equip them to provide high- quality pain treatment to their patients in line with World Health Organization (WHO) guidelines.<br>• Four teaching hospitals selected for pilot project (Based on their palliative care activity and utilization of oral morphine solution). Expanded to 15 by 2018
	National Comprehensive Cancer Network Harmonized Guidelines for Sub-Saharan Africa (2018 - 2019):	• Stakeholder workshops held leading to regional resources created as part of a collaborative efforts to combat rising cancer rates and unique care including palliative care
Uganda	Ministry of Health and National Drug clinical guidelines (2003):	• Nurses and Clinical officers are allowed to prescribe morphine for pain management
	Uganda Health Strategic plan, 2006–2011:	• Palliative Care is fully incorporated into the National Health Sector Strategic plan for the first time
	Evidence base for palliative care (2015)[48]	• A lack of policies and guidelines to support the development of palliative care was noted<br>• Plans to take forward palliative care development as part of the national health strategy
	Implementing a palliative care nurse leadership	• Trained palliative care nurses and clinical officers can effectively prescribe oral morphine

	fellowship program in Uganda (2016)[49]	
	Atlas of palliative care development (2017):	• Number of palliative care service outlets reported at 229<br>• Number of pediatric palliative care services reported at 2<br>• Has specialised training programmes for palliative care<br>• Palliative care services per million inhabitants reported at 5.87<br>• Has a Palliative Care National Association<br>• Oral morphine is produced locally
	National Cancer Control plan (2018):	• In the background based on an evidence brief for need for control strategy palliative care is recognised as part of the cancer control plan , and that it should be evidence based<br>• The Development of the NCCPs is underway, with a draft in place and palliative care is inclusive<br>• The NCCP addresses six key elements: (1) prevention, (2) early detection and screening, (3) diagnosis and treatment, (4) palliative care and (5) policy advocacy (6) research
	National eHealth strategy (2018):	• Baseline survey shows fragmented landscape of ICT pilot projects and numerous data and health information system (HIS) silos with significant barriers to the effective sharing of information between healthcare participants.<br>• Uganda adopts eHealth strategy to guide the use of ICT in supporting health sector transformation by addressing
Zimbabwe	WHO pilot study of palliative care in five African counties (2004): https://www.who.int/cancer/palliative/projectproposal/en/	• Estimated 1/60 people in Zimbabwe in need of palliative care<br>• Low level of palliative integration in the health system despite long history<br>• Training is a major strength<br>• Erratic pain relief
	African Palliative Care Association Policy and Gender Review in 10 countries (2010):	• Minimal coverage of palliative care<br>• No policies reviewed facilitated opioid availability<br>• Revealed gaps to be addressed to scale up integration of palliative care<br>• Recommended development of palliative care policy to guide actions and support for palliative care issues at national level

	National Palliative Care Situational Analysis commissioned by HOSPAZ (2012):	• Lack of knowledge and skills including listening and communication skills among care providers at all levels<br>• Need exists for palliative care training, bereavement counselling and home-based care<br>• Lack of knowledge / expertise in pain assessment and management using WHO analgesic ladder<br>• Unavailability and erratic supply of pain control medicines, especially strong opioids<br>• Children either not part of palliative care process or health providers have difficulty in handling their unique requirements (e.g. pain assessment and counselling)<br>• Poor integration of palliative care into public sector<br>• Lack of palliative care awareness and limited provision
	UNICEF and ICPCN Assessment of the Need for Palliative Care for Children: Three Country Report (2013):	• Number of children benefiting from palliative care services significantly lower than the estimated need (6% of specialised need)<br>• Lack of knowledge and adequate understanding of palliative care and children's palliative care among health professionals<br>• Lack of palliative care awareness by general public hence limited demand<br>• Poor pain management in children<br>• Morphine prescribing limited to medical doctors which is problematic
	African Palliative Care Association Morphine Survey (2015):	• Some progress toward integration of palliative care into health systems<br>• Advocacy for legal and policy changes recommended for nurse-prescribing of strong pain-relieving drugs, including opioids<br>• Opioid use in 2012 only 10.1kg instead of expected 177kg deduced from disease burden<br>• Recommended relevant knowledge on use of opioid analgesics to be integrated into medical and nursing school curricula
	Treat the Pain: A Country Snapshot (2016):	• Estimated 80% of cancer deaths (2012/2013) experience moderate to severe pain<br>• Average morphine consumption for 2011-2013 estimated 15.6kg, much lower than anticipated use<br>• Coverage of deaths in pain with treatment: 6%
