# Supplementary material for: Understanding data and information needs for palliative cancer care to inform digital health intervention development in Nigeria, Uganda and Zimbabwe: protocol for a multicountry qualitative study
Source: BMJ Open. 2019 Oct 31;9(10):e032166. doi: 10.1136/bmjopen-2019-032166 (PMC6830840; doi:10.1136/bmjopen-2019-032166)
Supplement: Supplementary data [file bmjopen-2019-032166supp002.pdf]

Appendix B: Facilities involved in identification and recruitment of participants

**Country**	**Facility name**	**Type of facility**	**Participants to recruit from facility**
Nigeria	Lagos University Teaching Hospital	Tertiary care facility	Patients, caregivers, health professionals
	Sebeccly Cancer Care and Support Center	Private not-for- profit, community organisation	Patients, caregivers, health professionals
	National Ministry of Health, Nigeria	National government office	Policymakers
Uganda	Uganda Cancer institute (UCI)	Tertiary care medical facility	Patients, caregivers, health professionals
	Makerere Palliative Care Unit, Mulago Hospital	National referral hospital	Patients, caregivers, health professionals
	Kawempe Home Care	Private not-for- profit, community organisation	Patients, caregivers, health professionals
	Hospice Africa Uganda	Private not-for- profit, organisation with three facilities across Uganda	Patients, caregivers, health professionals
	Uganda Ministry of Health	National government office	Policymakers
Zimbabwe	Chitungwiza Hospital	Central hospital	Patients, caregivers, health professionals
	Parirenyatwa Group of Hospitals	Central hospital	Patients, caregivers, health professionals
	National Ministry of Health staff	National government office	Policymakers
	Island Hospice and Healthcare	Private not-for- profit, community organisation	Patients, caregivers, health professionals
