# Supplementary material for: Understanding data and information needs for palliative cancer care to inform digital health intervention development in Nigeria, Uganda and Zimbabwe: protocol for a multicountry qualitative study
Source: BMJ Open. 2019 Oct 31;9(10):e032166. doi: 10.1136/bmjopen-2019-032166 (PMC6830840; doi:10.1136/bmjopen-2019-032166)
Supplement: Author's manuscript [file bmjopen-2019-032166.draft_revisions.pdf]

BMJ Open

BMJ Open is committed to open peer review. As part of this commitment we make the peer review history of every article we publish publicly available.

When an article is published we post the peer reviewers' comments and the authors' responses online. We also post the versions of the paper that were used during peer review. These are the versions that the peer review comments apply to.

The versions of the paper that follow are the versions that were submitted during the peer review process. They are not the versions of record or the final published versions. They should not be cited or distributed as the published version of this manuscript.

BMJ Open is an open access journal and the full, final, typeset and author-corrected version of record of the manuscript is available on our site with no access controls, subscription charges or pay-per-view fees (http://bmjopen.bmj.com).

If you have any questions on BMJ Open's open peer review process please email info.bmjopen@bmj.com

BMJ Open

Understanding data and information needs for palliative cancer care to inform digital health intervention development in Nigeria, Uganda and Zimbabwe: protocol for a multi-country qualitative study

Journal:	*BMJ Open*
Manuscript ID	bmjopen-2019-032166
Article Type:	Protocol
Date Submitted by the Author:	06-Jun-2019
Complete List of Authors:	Okunade, Kehinde ; University of Lagos College of Medicine, College of Medicine<br>Bashan Nkhoma, Kennedy; King's College London, Florence Nightingale Faculty of Nursing Midwifery and Palliative Care<br>Salako, Omolola ; Lagos State University Teaching Hospital, Department of Radiation Oncology<br>Akeju, David; University of Lagos, Department of Sociology<br>Ebenso, Bassey; University of Leeds, Nuffield Centre for International Health and Development;<br>Namisango, Eve; African Palliative Care Association<br>Soyannwo, Olaitan ; University College Hospital Ibadan<br>Namukwaya, Elizabeth; Makerere University College of Health Sciences, Palliative care unit, Department of Internal Medicine<br>Dandadzi, Adlight ; University of Zimbabwe College of Health Sciences, Clinical Trials Research Centre<br>Nabirye, Elizabeth; Makerere University College of Health Sciences, Palliative care unit, Department of Internal Medicine<br>Mupaza, Lovemore; Island Hospice and Healthcare<br>Luyirika, Emmanuel; African Palliative Care Association<br>Ddungu, Henry; Uganda Cancer Institute<br>Chirenje, Z. Mike ; University of Zimbabwe College of Health Sciences<br>Bennett, Michael; University of Leeds, Academic Unit of Palliative Care, Leeds Institute of Health Sciences<br>Harding, Richard; King's College London, Florence Nightingale Faculty of Nursing Midwifery and Palliative Care<br>Allsop, Matthew ; University of Leeds, Academic Unit of Palliative Care, Leeds Institute of Health Sciences
Keywords:	PALLIATIVE CARE, ONCOLOGY, Information technology < BIOTECHNOLOGY & BIOINFORMATICS, International health services < HEALTH SERVICES ADMINISTRATION & MANAGEMENT

For peer review only - http://bmjopen.bmj.com/site/about/guidelines.xhtml

Understanding data and information needs for palliative cancer care to inform digital health intervention development in Nigeria, Uganda and Zimbabwe: protocol for a multi-country qualitative study

Author list: Kehinde Okunade[1], Kennedy Bashan Nkhoma[2], Omolola Salako[3], David Akeju[4], Bassey Ebenso[5], Eve Namisango[6], Olaitan Soyannwo[7], Elizabeth Namukwaya[8], Adlight Dandadzi[9], Elizabeth Nabirye[8], Lovemore Mupaza[10], Emmanuel Luyirika[6], Henry Ddungu[11], Mike Chirenje[9], Michael I Bennett[5], Richard Harding[2], Matthew J Allsop[5]*.

Author affiliation:

[1] College of Medicine, University of Lagos, University of Lagos, Lagos, Nigeria

[2] Florence Nightingale Faculty of Nursing Midwifery and Palliative Care, Cicely Saunders Institute, Kings College London, UK

[3] Department of Radiation Oncology, Lagos University Teaching Hospital, Lagos State, Nigeria

[4] Department of Sociology, University of Lagos, University of Lagos, Lagos, Nigeria

[5] Academic Unit of Palliative Care, Leeds Institute of Health Sciences, University of Leeds, Leeds, UK

[6] African Palliative Care Association, Kampala, Uganda

[7] Centre for Palliative Care, University College Hospital, Ibadan, Nigeria

[8] Palliative care unit, Department of Internal Medicine, Makerere University, Kampala, Uganda

[9] Clinical Trials Research Centre, College of Health Sciences, University of Zimbabwe, Harare, Zimbabwe

[10] Island Hospice and Healthcare, Harare, Zimbabwe

[11] Uganda Cancer Institute, Kampala, Uganda

**Corresponding author:* Dr Matthew Allsop, Academic Unit of Palliative Care, Leeds Institute of Health Sciences, University of Leeds, Worsley Building Clarendon Way, LS2 9NL, Leeds, UK. Email: m.j.allsop@leeds.ac.uk. Phone: +44(0)113 343 4185.

Word count: 2,994 words

For peer review only

ABSTRACT

Introduction: Palliative care is a clinically and cost-effective component of cancer services in SSA. Despite the significant need for palliative cancer care in SSA, coverage remains inadequate. The exploration of digital health approaches could support increases in the quality and reach of palliative cancer care services in SSA. However, there is currently a lack of any theoretical underpinning or data to understand stakeholder drivers for digital health components in this context. This project addresses this gap through engaging with key stakeholders to determine data and information needs that could be supported through digital health interventions.

Methods and analysis: This is a multi-country, cross-sectional, qualitative study conducted in Nigeria, Uganda and Zimbabwe. In-depth interviews with patients with advanced cancer, caregivers, health professionals and policy makers will be used to understand the acceptability and define mechanisms of patient-level data capture and usage via digital technologies. This will determine optimal mechanisms for the design and development of subsequent digital health interventions to support the development, access and delivery of palliative cancer care in SSA.

Discussion: Primary data will guide subsequent piloting and evaluation of digital health interventions to improve cancer palliative care for patients and families. This could include the development of patient-focused digital health interventions such as information provision and self-management support, facilitate utilisation of patient data to prioritise allocation of scarce human resources, and exploration of the influence of factors such as gender, intersectionality, disability and cancer type on utilisation and engagement with digital health approaches.

Trial registration number: ISRCTN15727711

Key words: cancer, qualitative study, public health, health services research

For peer review only

For peer review only - http://bmjopen.bmj.com/site/about/guidelines.xhtml

ARTICLE SUMMARY

- This study is the first to identify preferences of stakeholders involved in palliative cancer care provision in sub-Saharan Africa to inform digital health approaches
- All aspects of the proposed study have been coproduced with experts in palliative care delivery and research in Nigeria, Uganda and Zimbabwe
- This multi-country study will generate a logic model to target digital health approaches for palliative care that could have relevance across the sub-Saharan Africa region
- This study restricts its focus to palliative cancer care so its relevance to other palliative conditions may be limited
- The findings may not be reproducible beyond the three participating countries.

For peer review only

INTRODUCTION

Due to late-stage clinical presentation, limited funding and restricted access to curative therapies, about 80% of cancers on the continent are incurable at the time of detection and diagnosis.[1] In 2018, there were over 770,000 new cancer cases and 514,000 cancer-related deaths across Eastern, Middle, Southern and Western Africa.[2] These figures are projected to continue to rise (up to 1.28 million new cases and 970,000 deaths) by 2030, with subsequent international and regional political declarations constituting a new global non-communicable disease agenda.[3] Increasing incidence is attributed to factors that include ageing, high residual burden of infectious agents (HIV/AIDS, human papillomavirus, hepatitis B virus) and lifestyle factors in sub-Saharan Africa (SSA).[4] Our analysis reveals that by 2060, an estimated 16 million people with cancer will die annually with serious health-related suffering, a 109% increase between 2016 and 2060, with the fastest rise occurring in low-income countries (400% increase).[5]

Palliative care (PC)—the prevention and relief of physical, emotional, social, or spiritual suffering associated with any chronic or life-threatening illness, from the point of diagnosis—is a vital and fundamental component of the basic and essential services within Universal Health Coverage (UHC).[6] It is also a realistic response to support equitable, accessible and cost-effective interventions for cancer care in SSA. Independent of cancer prevention and treatment efforts in the region, PC remains a critical and essential component of care, with proven effectiveness and cost effectiveness.[7, 8] There have been enormous strides made in the development of PC services in the SSA region[9, 10] but there remains a need for significant expansion of provision to meet demand. Current provision of PC services is limited to 24 of 48 countries, up from only five in 2004, with only less than 5% of people who need PC being able to access services in the region.[10]

A major challenge to developing palliative cancer care across the African region is the lack of local evidence to ensure practice is evidence-based and replicable and reflects the needs of the population served.[6] Evidence to date has revealed that advanced cancer patients in SSA have a high burden of physical and psychological symptoms, would prefer to have full information and better communication around their needs and care options, experience spiritual distress, their family caregivers face compounded poverty and psychological distress.[11-18] It is essential to create channels for gathering patient-level data as an indicator of quality as well as to inform clinical practice and audit. Furthermore, understanding how emerging services are supporting patients with advanced cancer through assessing experiences and outcomes is a priority for PC development in the region. [19] This can be achieved through the use of validated, context-specific tools for measuring outcomes

For peer review only - http://bmjopen.bmj.com/site/about/guidelines.xhtml

for PC patients and their caregivers in SSA.[19, 20] We have therefore developed a valid patient-reported outcome measure (PROM) for advanced disease in SSA to capture the core concerns of patients and families.[21-23] Capturing these data can enable development and adaptation of services to ensure they can respond to the specific needs of patients with cancer.

This protocol describes a study to explore how technology-based approaches could capture patient-level data from patients with advanced cancer that has utility across the health system. Previous work by our team has highlighted the potential of digital health to facilitate the collection, sharing and use of patient-level data. For example, we know that mobile phones are frequently used in multiple ways as part of PC service provision in the African region and that development of approaches that capitalise on mobile phones is a high priority for providers.[24] In SSA, mobile phone services are available to a larger portion of the population than many basic services (such as sanitation and financial services). Approaches using digital health can benefit from the widespread access and low cost of mobile phone devices in the region and have shown improved chronic disease management;[24] patient behaviour change and health systems strengthening;[25] reduced costs of patient monitoring; improved adherence; and better communication. These benefits are greatest in rural areas[26]. Furthermore, such patient to provider telemedicine has recently been recommended by the World Health Organization (WHO) as an approach that can support health systems strengthening.[27]

In recent years, there has been exploratory research and development of digital health approaches in PC services in SSA.[28-30] However, this project seeks to address the lack of a theoretical underpinning to interventions using digital health components in this context. Our project will undertake engagement with key stakeholders (patient, caregivers, health professionals and policymakers) across the health system to define the optimal mechanisms through which patient-level data, captured via digital health approaches, can be integrated into palliative cancer care delivery and improvement.

Research objectives and questions

The study aims to answer the question, what are the optimal mechanisms through which patient-level data, captured via digital health, can be used in the development and delivery of palliative cancer care in sub-Saharan Africa?

The specific objectives of the project are to:

1. Establish a consortium of academic researchers (from the UK, Nigeria, Uganda, and Zimbabwe), service user advocates, non-governmental organisations, palliative care

For peer review only - http://bmjopen.bmj.com/site/about/guidelines.xhtml

providers, policymakers and digital health development and implementation experts, to catalyse digital health research and generate evidence that can guide palliative cancer care development across sub-Saharan Africa.

2. Understand the acceptability and optimal implementation of patient-level data collection (e.g. patient-reported outcome measures and patient-reported experience measures) using digital health approaches in Uganda, Nigeria and Zimbabwe through patient and caregiver engagement.
3. Determine information needs and pathways for leveraging evidence generated from digital health approaches in service development in Uganda, Nigeria and Zimbabwe through health professional and service manager engagement.
4. Determine information needs and pathways for leveraging evidence generated from digital health approaches in policymaking in Uganda, Nigeria and Zimbabwe through policymaker engagement.
5. Define the mechanisms for implementation of digital health approaches to support development of palliative cancer care in sub-Saharan Africa.
6. Develop a theoretically-informed logic model for implementing digital health approaches to improve palliative care in sub-Saharan Africa.

METHODS AND ANALYSIS

Through partnership with the African Palliative Care Association (APCA), the University of Leeds have assessed the use and priorities of digital health approaches in PC services in the African region.[28] Furthermore, Kings College London, working with APCA, have been pioneering patient-level data collection in PC in SSA and developing Patient Reported Outcome Measures (PROMS);[19, 20] simple checklists of symptoms and concerns, that are widely adopted and enable staff, patients and families to identify main concerns and prevent suffering, maintain people at home, support families, and optimise function. PROMS for people with serious incurable illness can improve care and patient wellbeing. This project will enable integration of these research initiatives, determining the architecture of digital technologies to facilitate uptake and utilisation of evidence-based approaches such as PROMS. This will take forward the science of digital health in this neglected field, enabling a logic model to be developed for subsequent evaluation and implementation.

Palliative care development in participating countries

The current development of PC for each participating country is summarised in Table 1.

For peer review only - http://bmjopen.bmj.com/site/about/guidelines.xhtml

Table 1: Summary of palliative care development in Nigeria, Uganda and Zimbabwe

**Country**	**Summary of development**
Nigeria	In Nigeria – the most populous country in SSA – palliative care (PC) is disparately spread in centres across the country. Historically, PC development in western Africa has been secondary to developments in the eastern and southern parts of the continent, and this continues to be the case. This is due mainly to the relatively low HIV prevalence, which meant that the region did not qualify to receive funding from the US President's Emergency Plan for AIDS Relief (PEPFAR) in the early 2000s.[31] Widespread interest in palliative care development in Nigeria emanated from the pain and suffering witnessed by concerned health professionals in patients with advanced cancer. Early efforts by the Hospice Nigeria team included an advocacy visit by Anne Merriman (the founder of Hospice Africa Uganda in 1993) which yielded little result as there was no opioid analgesics in the country to manage the associated cancer pain.[32] The current movement that has resulted in establishment of holistic palliative care services across Nigeria commenced in 1996 with the Ibadan "Cancer pain group".[33] Concerted advocacy and other activities of the group facilitated importation of opioid analgesics by the Federal Ministry of Health for management of severe pain by 2001 and morphine powder for oral morphine preparation by 2005.<br>The first PC team was founded in 2003 at Ibadan, providing both hospital- and home-based services,[31] and the Hospice and PC Association of Nigeria was created in 2007. Members, most of whom received palliative care initiator's training at Hospice Africa, Uganda function as individuals or teams across the six geopolitical areas of the country. Home-based PC continues as a very important form of PC delivery, with evidence of its benefits to patients and their families.[34] Some aspects of palliative care are now included in the national guidelines for HIV and AIDS treatment and care in adolescents and adults (2012) as well as the national cancer control plan of the FMOH (2018-2022)<br>Current barriers to PC development include lack of government guidelines, poor knowledge about PC importance at all levels – policy makers, public, health professionals, lack of inclusion of palliative care in curricula of health professionals and in the national health budget and national health Insurance (NHIS), poor availability and accessibility of strong opioid analgesics for cancer pain management. However, since 2012, the 'Treat Pain project' and "Pain Free

For peer review only - http://bmjopen.bmj.com/site/about/guidelines.xhtml

	Hospital Initiative" of the Federal Ministry of Health, Global Access to Pain Relief Initiative (GAPRI) and American cancer Society have improved the opioid situation.[35, 36] More can still be achieved through the training of more health professionals and change of attitude, increased funding, and increased public awareness of the services.[34] There has been some early context setting work suggesting willingness to explore the application of technology within PC services, such as the use of telemedicine.[37]
Uganda	The Atlas of Palliative Care Development in Africa ranked Uganda highly in terms of palliative service development and its integration into the health system.[38] Prior to this, The Economist intelligence unit quality of death index[39] which is a "measure of the quality of palliative care provided to adults in over 80 countries", ranked Uganda as 35th in the world. This index gives a general impression of the quality of life at the end of life and recognises continued development of PC services in Uganda. In terms for service delivery, Uganda has over 229 palliative care service outlets, for a population of 40 million people. These include hospices, home-based care and health facility-based outlets. The level of pediatric palliative care service development remains poor, with two pediatric palliative care service centres despite a large population of children and young people.[1]<br>In terms of the wider health system, Uganda has been progressing the presence and development of palliative care services. Uganda is building a critical mass for palliative care professionals with palliative care integrated into the medical and nursing and is a hub for training and education in palliative care, with diploma, certificate and degree courses in palliative care provided by universities, specialised palliative care institutions such as Mildmay Uganda, and international organisations such as the African Palliative Care Association. In terms of health information systems, there are two national indicators of palliative care into the national electronic health information system; patients presenting with pain and those receiving morphine for pain management. With this advancement, to some extent, performance of palliative care can be monitored at the national level.<br>Access to pain medication is a crucial component of palliative care delivery in Uganda. The availability of the full analgesic ladder and palliative medicines remains poor,[40] compromising care providers' ability to support management of symptoms and alleviate suffering associated with pain. However, Uganda has introduced prescribing across cadres other than doctors, such as palliative care

For peer review only - http://bmjopen.bmj.com/site/about/guidelines.xhtml

	nurse prescribers. Furthermore, the rolling out of local production of morphine within Hospice Africa Uganda led to significant reductions in costs associated with morphine procurement.[41] This key opioid for moderate to severe pain is now provided free of change and is supplied through the national supply chain mechanisms of the country. Although oral morphine is free, access still remains a challenge because of several factors such frequent stock outs, reluctance to prescribe morphine for fear of addictions as well as limited access to this medicine at primary health care level.[29]<br>Palliative care is included in the national health strategic plan, and in the HIV care guidelines. A standalone palliative care policy was drafted in 2017 and is pending cabinet approval. It is envisioned that a standalone policy will be a strong pillar in giving a strategic direction to service development and evaluation to provide a comprehensive framework for the integration of palliative care services into the health care system. It will also be used as tool to advance financing for palliative care advocacy.
Zimbabwe	In 2014, a WHO report identified 1 in 60 Zimbabweans need palliative care,[42] alongside an International Children's Palliative Care Network report identifying a significant need among children across multiple provinces in Zimbabwe.[43] Despite the continuing high level of palliative care need, Zimbabwe was the first country in sub-Saharan Africa to have a hospice, founded in 1979. In terms of development of palliative care across the country, by 1997 there were 17 regional branches that had been formed throughout the country with 13 organisations providing palliative care by 2004.[44] The disease focus of palliative care in Zimbabwe was initially cancer, although the growth of the disease burden due to HIV and AIDS led to widening of provision to include those living with HIV and AIDS and other chronic illnesses. Notable initiatives have facilitated palliative care services provision in the country. In 1992, the Ministry of Health and Child Care formed the Prevention and Control of Cancer Committee in Zimbabwe that comprised relevant stakeholders and professionals. The committee oversaw the development of a ten-year National Cancer Control Programme Plan for Zimbabwe (1994 – 2004), with the overall aim to formulate, plan and implement a coordinated and cost-effective programme for the prevention and control of cancer in Zimbabwe. This led to the establishment of palliative care training across eight provinces and two cities of Harare and Bulawayo. The African Palliative Care

For peer review only - http://bmjopen.bmj.com/site/about/guidelines.xhtml

	Association (APCA) in 2010 reviewed national policy documents and implementation guidelines from ten Southern African countries, including Zimbabwe[45] For Zimbabwe, whilst palliative care was highlighted as a priority area across documents, issues around minimal coverage of palliative care was noted alongside an absence of detail relating to opioid availability. In more recent years the Worldwide Hospice Palliative Care Association (WHPCA) global update of mapping levels of palliative care provision in 2014, placed Zimbabwe in category 4a.[46] Category 4a suggests a country as hospice-palliative care services at a stage of preliminary integration into mainstream service provision. This category suggests the development of a critical mass of palliative care activism in a number of locations, a variety of palliative care providers and types of services, awareness of palliative care on the part of health professionals and local communities, the availability of morphine and some other strong pain-relieving medicines, limited impact of palliative care upon policy, the provision of a substantial number of training and education initiatives by a range of organisations, and interest in the concept of a national palliative care association.

In recent years, policy reports of PC from participating study countries have commonly identified priority focus areas of improving access to pain medications, improving awareness by health professionals of the value and role of PC in supporting patients, and integration of PC with the existing public health system. A summary of policy documents and their key findings for each country is provided in Appendix A.

For peer review only

Study design

This study will adopt a multi-country, cross-sectional, exploratory study using qualitative methods. In-depth interviews with patients, caregivers, health professionals and policy makers will be used to understand the acceptability and define mechanisms of patient-level data capture and usage. The interpretation of the findings will define optimal mechanisms through which patient-level data, captured via digital health, can be used in the development, delivery and improvement of palliative cancer care in sub-Saharan Africa. Alongside the planned research activities, a consortium focused on digital technology for palliative cancer care will be formed. This will include researchers from SSA and the UK, alongside key stakeholders in each of the participating countries (i.e. relevant policy representatives from ministries of health, civil society, patient advocates, and digital health specialists) to understand: i) current digital health development in Uganda, Nigeria and Zimbabwe; ii) opportunities for capacity development around digital health in palliative cancer care, and; iii) routes to uptake and translation of findings from planned research activities.

Study participants

The study participants will be adults living with advanced cancer, their caregivers, their health professionals, and policymakers with a focus on cancer, non-communicable diseases and/or technology. Patients will include adults with advanced cancer receiving palliative care. Caregivers will include those supporting palliative cancer patients receiving care from recruiting facilities. Health professionals will be drawn from the clinical teams associated with study partner institutes alongside related services delivered in the locality. Policymakers will comprise district or national level policymakers working within cancer, non-communicable diseases, or digital health. Table 2 outlines the inclusion / exclusion criteria, sampling characteristics and sample size for the four stakeholder groups.

For peer review only - http://bmjopen.bmj.com/site/about/guidelines.xhtml

Table 2: Population and sampling criteria for the four stakeholder groups

Participant group	Eligibility criteria	Sampling criteria	Target number to recruit in each participating country
**Patient**	***Inclusion criteria:***<br>• Patients with advanced cancer (defined as those with metastatic cancer (where, if possible, determined through histological, cytological or radiological evidence) and/or those receiving anti-cancer therapy with palliative intent) receiving palliative care<br>• Aged ≥ 18 years<br>***Exclusion criteria:***<br>• Patients with significant cognitive impairment that prevents informed consent<br>• Inadequate physical and mental health of a patient prior to recruiting (as deemed by the identifying clinician)<br>• Lack of a shared language between researcher and respondent<br>• Below 18 years of age	• Age<br>• Sex<br>• Cancer type<br>• Location at time of interview (community-based or ward-based)	• 20 purposely selected patients
**Caregiver**	***Inclusion criteria:***<br>• A primary caregiver/family caregiver of a patient with advanced cancer who is at least 18 years of age<br>• Primary caregiver confirmed by the patient, including those who provide unpaid, informal provision of one or more physical, social, practical and emotional tasks. In terms of their relationship to the	• Sex<br>• Age<br>• Patient involvement in study (ensuring representation of those where the patient whom they care for has participated,	• 15 purposely selected caregivers

For peer review only - http://bmjopen.bmj.com/site/about/guidelines.xhtml

	patient, they may be a friend, partner, ex-partner, sibling, parent, child or other blood or non-blood relative<br>• Both caregivers of participating and non-participating patients will be recruited into the study<br>***Exclusion criteria:***<br>• Below 18 years of age<br>• Inadequate physical and mental health of a patient prior to recruiting (as deemed by the identifying clinician)	and those where the patient has not participated).	
**Health professional**	***Inclusion criteria:***<br>• Working with palliative care patients<br>• At least six months working experience at recognised palliative care facilities	• Role (doctor, clinical officer, nurse, social worker, psychologist, pharmacist)<br>• Typical work setting (i.e. community-based, ward-based)	• 20 purposely selected health professionals
**Policymaker**	***Inclusion criteria:***<br>• Representative from government ministry or national association responsible for oversight and development of healthcare in participating country	• Working at different levels of the health system (district, national)<br>• Policy remit of their post (cancer, non-communicable diseases, digital health)	• Up to 10 purposely selected policymakers

For peer review only - http://bmjopen.bmj.com/site/about/guidelines.xhtml

Data collection and management

Patients

Clinical staff at recruiting facilities will be responsible for identifying participants. These staff will be asked to verify that patients are aware of their palliative care diagnosis. This will be through review of clinical records and discussion with the participant's health professionals by the research assistant. Patients who are deemed ethically inappropriate by members of the clinical team, for example where death is imminent, will not be approached. Participating patients will only be asked to participate in one face-to-face interview. The location of the interview will be decided by the patient or caregiver (i.e. meeting either at their home, following a clinic appointment at a health facility, on the ward, or at a neutral location, dependent on the patient's preference and the patient's clinical management at the time of the interview). Independent of the location chosen, a quiet and private room will be recommended to the patient for the interview. Arrangements will be made for such space at clinics and hospices ahead of interviews. A topic guide will be used to direct semi-structured interviews with patients. The topic guide will explore current interaction with and access to PC services, their use of technology, the acceptability of using digital technology approaches to support interaction with health services and data collection, the clinical response anticipated from health services (with and without facilitation by digital health interventions) including perceptions of effective responses, and alternative approaches to patient-level data collection without mobile phones.

Caregivers

Caregivers of patients with advanced cancer will be identified and approached to participate by clinical staff at recruiting facilities. Caregivers will only be required to participate in one face-to-face interview. A topic guide will be used to direct semi-structured interviews with caregivers. The themes addressed in the topic guide will align with the patient topic guide.

Healthcare professionals

Health professionals will be identified by clinical leads in each of the three countries. Through existing networks of PC providers in each of the countries, the clinical lead will approach health professionals to participate in the study. A topic guide will be used during health professional interviews. The topic guide aligns with stages of the data-use conceptual framework[15]: data demand (e.g. current availability, use and quality of data for clinical decision making), data collection (e.g. feasibility of digital technology approaches to patient-level data collection), data availability (e.g. clinical triggers in management of patients with advanced cancer, capacity to respond to information, information needs to inform patient care), and data utilization (e.g. sharing and accessing data via digital health

For peer review only - http://bmjopen.bmj.com/site/about/guidelines.xhtml

approaches, data reporting priorities to regional and national health authorities). Causal elements linked to organisational, technical and behavioural factors influencing data use will be explored for each stage.

Policymakers

Policymakers will be identified and approached by the African Palliative Care Association alongside academic and clinical teams in Nigeria, Uganda and Zimbabwe. A topic guide for key informant interviews will address access to and use of evidence to inform decision making, seek comment on findings from a desktop review of existing policy on digital health to be conducted prior to key informant interviews, preferred mode and presentation of data, frequency of data reporting needed to inform decision-making on financing of PC services and on accelerating universal health coverage.

Interviews across all recruitment sites in Uganda, Nigeria and Zimbabwe (outlined in Supplementary Appendix B) will be undertaken by research assistants, supervised by the academic partners in Nigeria (KO), Uganda (LN) and Zimbabwe (MC). All interviews will be audio recorded. Data security will be ensured through use of password-protected file sharing using the Microsoft OneDrive platform. Separate folders will be created for each country, with oversight from the lead institution, the University of Leeds. Only research team members will have access to the folder. All members of the project team will sign a data sharing agreement outlining explicit guidance regarding handling and management of research data that takes account of both the funders and national research council guidelines. On completion of this study, all electronic data on the OneDrive folder will be moved to an electronic archive for five years prior to being permanently destroyed. De-identified research data deemed suitable for sharing will be hosted by Research Data Leeds, the institutional research data repository for the University of Leeds. All study participants will be assigned an identification code, which will be delinked from their identity at data entry point.

Reflectivity

The research team comprises experts in digital health intervention development (MA, BE), qualitative research (DA, AD, EN), global PC provision (KBN, EN, EL, RH), monitoring and evaluation (LM), health services research in SSA (MC) alongside consultants in palliative medicine (EN, HD, OS, MB) and oncologists (KO, OS). None of these researchers have any relationship with the patients or caregivers who will be approached to participate. However, a small number of the healthcare professionals or policymaker participants may be known contacts.

For peer review only - http://bmjopen.bmj.com/site/about/guidelines.xhtml

Patient and public involvement

The rationale for this work arose from priorities identified through surveying a key stakeholder group, healthcare providers delivering palliative care across sub-Saharan Africa.[24] Patients and caregivers were not involved in the design of the study. However, alongside research activities, the team will develop a consortium which will include patient advocates. This forum will be used to explore routes for communicating study findings to patient groups and help to establish potential routes for identifying patient and caregivers, or advocates, in subsequent projects.

Data Analysis

Interviews will be transcribed verbatim and translated into English before being imported into NVivo software for deductive framework analysis.[47] An initial charting of pseudonymised transcripts (KN) will be developed through line-by-line coding. This initial code will then be applied to a random set of transcripts (n=3) in each stakeholder group by three further members of the research team (Nigeria, Uganda, Zimbabwe), and the final framework agreed through discussion with the wider team. Once inconsistencies are resolved, the joint coding frame will be used to code all interviews across the participant group. Comparative analysis in the framework will enable us to identify common themes as well as country-specific and stakeholder group divergences. A model of the coding frame will be developed, and each theme and subtheme given a definition to ensure internal consistency of each code. Illustrative codes will be reported for each theme, with the study ID code to demonstrate reporting from across the sample breadth. In the final project meeting we will hold a Theory of Change workshop to model the planned digital health pathway within its context, detailing processes, stakeholder roles, outcomes and intended impact.

Ethical considerations

Ethics approvals have been obtained from the Institutional Review Boards of University of Leeds (MREC 18-032), Research Council of Zimbabwe (Ref: 03507), Medical Research Council of Zimbabwe (Ref: MRCZ/A/2421), Uganda Cancer Institute (UCI REC Ref: 19-2018), Uganda National Council of Science and Technology (Ref: HS325ES), and College of Medicine University of Lagos (HREC/15/04/2015). Ethical review undertaken by all project investigators ensured standard processes (dignity, autonomy, informed consent, confidentiality, anonymity, ability to adhere to protocol) and data security were considered in the protocol development. The project will be aligned with the MRC good research practice guidelines and H3Africa framework for conducting ethically responsible biomedical research. With reference to research on palliative care populations, a patient's condition should not preclude them from participation. Instead, additional strategies of harm

For peer review only - http://bmjopen.bmj.com/site/about/guidelines.xhtml

minimisation have been developed collaboratively by the research team. Academic leads and co-investigators will support protocol adaptation for local cultural appropriateness (e.g. modifying recruitment accounting for cancer-related stigma). Researchers will routinely contact clinical teams to check the health status of a patient prior to contact for research activities. In terms of data governance, project documentation and de-identified data for joint analysis will be shared via a secure IT infrastructure hosted by the University of Leeds. Voluntary and informed participation, confidentiality and safety of participants will constitute key principles of researcher–respondent interaction. Written consent or a thumb print will be obtained from patients, caregivers, health care professionals and policymakers prior to their enrolment in the study.

DISSEMINATION

We will provide an understanding of the mechanisms by which digital health approaches can facilitate evidence generation and use, ensuring optimal implementation with clear pathways for integrating captured data into existing health system functions. On completion of planned research activities, we will have:

1. Instigated an active, interdisciplinary consortium focused on technology-based approaches to developing palliative cancer care in SSA;
2. Defined mechanisms for optimal implementation of digital health interventions to support PC service development in SSA;
3. Provided a list of factors to target with digital health approaches with accompanying programme theory;
4. Obtained essential preliminary data needed to inform future research in digital health technology development for PC in SSA;
5. Developed a logic model for implementation of digital health to improve advanced cancer care in SSA.
6. Strengthened capacity for research, innovation and knowledge exchange in partner institutions and identified future capacity building needs.

Undertaking primary research with patients, caregivers, health professionals and policymakers is essential to guide digital health approaches for palliative cancer care in SSA. A number of activities will be undertaking to support dissemination of project findings. These will include: i) developing newsletters and press-releases to communicate key project findings to the general public; ii) developing a dedicated website for the study where results will be publicly accessible by national and international policymakers, practitioners and academics; iii) delivering presentations at local and

For peer review only - http://bmjopen.bmj.com/site/about/guidelines.xhtml

national conferences in participating countries, alongside presentation at international conferences; iv) publishing articles in peer-reviewed journals; and v) social media through research team member and institutional accounts. Participants will be anonymised in any dissemination activities. Only pseudonymised, non-identifiable characteristics and quotes will be used in dissemination.

CONCLUSION

This paper reports the protocol for a cross-sectional study with qualitative methods aimed at understanding the optimal mechanisms through which patient-level data, captured via digital health, can be used in the development, access and delivery of quality palliative cancer care in Uganda, Nigeria and Zimbabwe. The findings of the study will be device agnostic, providing a theoretical framework that can be used to inform a wide range of digital health intervention development and implementation. Without this research there is a risk of digital health intervention development for PC occurring in silos across SSA that do not take account of the multiple uses and value of data for stakeholders across the wider health system. Mapping information and data needs across PC services will also create multiple opportunities for research. This includes subsequent piloting and evaluation of digital health interventions and validation of the data they capture, development of patient-focused digital health interventions such as information provision and self-management support, and exploration of the influence of factors such as gender, intersectionality, disability and cancer type on utilisation and engagement with digital health approaches. Subsequent development of digital health approaches for PC in SSA, gathering patient-level data and facilitating patient-provider communication, could lead to multiple benefits for patients and caregivers (reduced costs associated with time and travel to facilities, extend coverage and reach of services such as rural areas with mobile connectivity), health professionals (ability to identify and respond to specific and rising demand from patients with cancer), and policymakers (receiving appropriate and timely data to inform service planning, guide integration of PC with wider healthcare delivery, and contribute to strengthening of national digital health systems). There is an added imperative to understand how best to utilise digital health technologies for those receiving and providing care for advanced disease in SSA. There is currently a lack of evidence on the preferences of patients with advanced disease, their caregivers and their health professionals. Understanding these preferences is crucial to inform digital health intervention development, such as modelling interventions around the preferences of the timing and delivery of digital health approaches in PC.

For peer review only - http://bmjopen.bmj.com/site/about/guidelines.xhtml

PROTOCOL AND REGISTRATION

This study protocol is registered on the ISRCTN website (ISRCTN15727711).

ACKNOWLEDGEMENTS

We would like to thank Olasupo Oyedepo, Director of the African Alliance of Digital Health Networks and Project Director at ICT4HEALTH Project for guidance on the development and implementation of this project.

CONTRIBUTORS

MA conceived the study; MJA, BE, RH, EN (Namisango), EN (Namukwaya) and MC contributed to the development of the study design and final protocols for sample selection, observations and interviews; KO, MJA, OS and EN (Namisango) developed a draft of the manuscript; All authors (MJA, KO, KBN, OS, DA, BE, EN, OS, EN, AD, EN, LM, EL, HD, MC, MIB, RH) contributed to writing the manuscript.

FUNDING

This work was supported by the Medical Research Council (grant number MR/S014535/1) and Research England Quality-related Global Challenges Research Fund (QR GCRF) through the University of Leeds.

COMPETING INTERESTS

None declared.

ETHICS

Ethics approvals have been obtained from the Institutional Review Boards of the University of Leeds (MREC 18-032), Research Council of Zimbabwe (Ref: 03507), Medical Research Council of Zimbabwe (Ref: MRCZ/A/2421), Uganda Cancer Institute (UCI REC Ref: 19-2018), Uganda National Council of Science and Technology (Ref: HS325ES), and College of Medicine at the University of Lagos (HREC/15/04/2015).

For peer review only - http://bmjopen.bmj.com/site/about/guidelines.xhtml

DATA SHARING STATEMENT

No additional data available.

PROVENANCE AND PEER REVIEW

Not commissioned; externally peer reviewed.

For peer review only

For peer review only - http://bmjopen.bmj.com/site/about/guidelines.xhtml

REFERENCES

1. Ferlay, J., I. Soerjomataram, R. Dikshit, et al. *Cancer incidence and mortality worldwide: sources, methods and major patterns in GLOBOCAN 2012.* International journal of cancer, 2015. **136**(5): p. E359-86.
2. International Agenct for Research on Cancer, *Globocan: All cancers*. 2018, International Agenct for Research on Cancer: France.
3. Powell, R.A., Z. Ali, E. Luyirika, et al. *Out of the shadows: non-communicable diseases and palliative care in Africa.* BMJ Supportive & Palliative Care, 2015.
4. Jemal, A., P. Vineis, F. Bray, et al., *The Cancer Atlas*. 2016, Atlanta, Georgia: American Cancer Society.
5. Sleeman, K., M. Brito, S. Etkind, et al. *The escalating global burden of serious health-related suffering: projections to 2060 by world regions, age groups and health conditions. .* Lancet Global Health, 2019. **In Press**.
6. Knaul, F.M., P.E. Farmer, E.L. Krakauer, et al. *Alleviating the access abyss in palliative care and pain relief—an imperative of universal health coverage: the Lancet Commission report.* The Lancet, 2018. **391**(10128): p. 1391-454.
7. Gomes, B., N. Calanzani, V. Curiale, et al. *Effectiveness and cost-effectiveness of home palliative care services for adults with advanced illness and their caregivers.* Cochrane Database of Systematic Reviews, 2013(6).
8. Smith, S., A. Brick, S. O'Hara, et al. *Evidence on the cost and cost-effectiveness of palliative care: a literature review.* Palliat Med, 2014. **28**(2): p. 130-50.
9. Fraser, B.A., R.A. Powell, F.N. Mwangi-Powell, et al. *Palliative Care Development in Africa: Lessons From Uganda and Kenya.* Journal of Global Oncology, 2017(4): p. 1-10.
10. Downing, J., L. Grant, M. Leng, et al. *Understanding Models of Palliative Care Delivery in Sub-Saharan Africa: Learning From Programs in Kenya and Malawi.* Journal of Pain and Symptom Management, 2015. **50**(3): p. 362-70.
11. Beynon, T., E. Radcliffe, F. Child, et al. *What are the supportive and palliative care needs of patients with Cutaneous T cell Lymphoma (CTCL) and their caregivers? A systematic review of the evidence.* The British journal of dermatology, 2013.
12. Harding, R., L. Selman, G. Agupio, et al. *The prevalence and burden of symptoms amongst cancer patients attending palliative care in two African countries.* European journal of cancer, 2011. **47**(1): p. 51-6.
13. Selman, L., I.J. Higginson, G. Agupio, et al. *Meeting information needs of patients with incurable progressive disease and their families in South Africa and Uganda: multicentre qualitative study.* Bmj, 2009. **338**: p. b1326.
14. Selman, L.E., I.J. Higginson, G. Agupio, et al. *Quality of life among patients receiving palliative care in South Africa and Uganda: a multi-centred study.* Health Qual Life Out, 2011. **9**(1): p. 21.
15. Selman, L., R. Harding, I. Higginson, et al. *Spiritual wellbeing in sub-Saharan Africa: the meaning and prevalence of 'feeling at peace'.* BMJ Support Palliat Care, 2011. **1**(Suppl 1): p. A22.
16. Selman, L., R.J. Siegert, I.J. Higginson, et al. *The MVQOLI successfully captured quality of life in African palliative care: a factor analysis.* Journal of clinical epidemiology, 2011. **64**(8): p. 913-24.
17. Selman, L., R.J. Siegert, I.J. Higginson, et al. *The "Spirit 8" successfully captured spiritual well-being in African palliative care: factor and Rasch analysis.* Journal of clinical epidemiology, 2012. **65**(4): p. 434-43.
18. Streid, J., R. Harding, G. Agupio, et al. *Stressors and resources of caregivers of patients with incurable progressive illness in sub-Saharan Africa.* Qualitative health research, 2014. **24**(3): p. 317-28.

For peer review only - http://bmjopen.bmj.com/site/about/guidelines.xhtml

19. Bausewein, C., B.A. Daveson, D.C. Currow, et al. *EAPC White Paper on outcome measurement in palliative care: Improving practice, attaining outcomes and delivering quality services – Recommendations from the European Association for Palliative Care (EAPC) Task Force on Outcome Measurement.* Palliative Medicine, 2015. **30**(1): p. 6-22.
20. Harding, R., L. Selman, G. Agupio, et al. *Validation of a core outcome measure for palliative care in Africa: the APCA African Palliative Outcome Scale.* Health Qual Life Out, 2010. **8**: p. 10-10.
21. Blum, D., L.E. Selman, G. Agupio, et al. *Self-report measurement of pain & symptoms in palliative care patients: a comparison of verbal, visual and hand scoring methods in Sub-Saharan Africa.* Health Qual Life Outcomes, 2014. **12**(1): p. 118.
22. Harding, R., L. Selman, G. Agupio, et al. *Validation of a core outcome measure for palliative care in Africa: the APCA African Palliative Outcome Scale.* Health and Quality of Life Outcomes, 2010. **8**.
23. Harding, R., L. Selman, V.M. Simms, et al. *How to analyze palliative care outcome data for patients in Sub-Saharan Africa: an international, multicenter, factor analytic examination of the APCA African POS.* J Pain Symptom Manage, 2013. **45**(4): p. 746-52.
24. Beratarrechea, A., A.G. Lee, J.M. Willner, et al. *The Impact of Mobile Health Interventions on Chronic Disease Outcomes in Developing Countries: A Systematic Review.* Telemedicine and e-Health, 2013. **20**(1): p. 75-82.
25. Peiris, D., D. Praveen, C. Johnson, et al. *Use of mHealth Systems and Tools for Non-Communicable Diseases in Low- and Middle-Income Countries: a Systematic Review.* J. of Cardiovasc. Trans. Res., 2014: p. 1-15.
26. Betjeman, T.J., S.E. Soghoian, and M.P. Foran. *mHealth in sub-Saharan Africa.* Int. J. Telemedicine Appl., 2013. **2013**: p. 6-6.
27. World Health Organization, *Recommendations on digital interventions for health system strengthening*. 2019, World Health Organization: Geneva, Switzerland.
28. Allsop, M.J., E. Namisango, and R.A. Powell. *A survey of mobile phone use in the provision of palliative care services in the African region and priorities for future development.* Journal of Telemedicine and Telecare, 2018. **25**(4): p. 230-40.
29. Namisango, E., C. Ntege, E.B.K. Luyirika, et al. *Strengthening pharmaceutical systems for palliative care services in resource limited settings: piloting a mHealth application across a rural and urban setting in Uganda.* BMC Palliative Care, 2016. **15**(1): p. 20.
30. Allsop, M.J., R.A. Powell, and E. Namisango. *The state of mHealth development and use by palliative care services in sub-Saharan Africa: a systematic review of the literature.* BMJ Supportive & Palliative Care, 2016: p. bmjspcare-2015-001034.
31. Ajayi, I., O. Iken, R.A. Powell, et al. *Palliative care research in western Africa.* European Journal of Palliative Care, 2014. **21**(1): p. 45-47.
32. Merriman, A., *Audacity to Love: The Story of Hospice Africa*. 2010, Ireland: Irish Hospice Foundation.
33. Soyannwo, O. *Hospice and palliative care development in West Africa sub region: A review.* African Journal of Anaesthesia and Intensive Care, 2013. **13**: p. 10-13.
34. Omoyeni, N., O. Soyannwo, O. Aikomo, et al. *Home-based palliative care for adult cancer patients in Ibadan-a three year review.* Ecancermedicalscience, 2014. **8**: p. 490.
35. O'Brien, M., F. Mwangi-Powell, I.F. Adewole, et al. *Improving access to analgesic drugs for patients with cancer in sub-Saharan Africa.* The Lancet. Oncology, 2013. **14**(4): p. e176-82.
36. O'Brien, M., A. Schwartz, and L. Plattner. *Treat the Pain Program.* Journal of Pain and Symptom Management, 2018. **55**(2): p. S135-S39.
37. van Gurp, J., O. Soyannwo, K. Odebunmi, et al. *Telemedicine's Potential to Support Good Dying in Nigeria: A Qualitative Study.* PloS one, 2015. **10**(6): p. e0126820.
38. Rhee, J.Y., E. Luyirika, E. Namisango, et al., *APCA Atlas of Palliative Care in Africa*. 2017, Texas, USA: IAHPC Press.

For peer review only - http://bmjopen.bmj.com/site/about/guidelines.xhtml

39. The Economist Intelligence Unit, *The 2015 Quality of Death Index: Ranking palliative care across the world* 201, The Economist: London, UK.
40. Harding, R., V. Simms, S. Penfold, et al. *Availability of essential drugs for managing HIV-related pain and symptoms within 120 PEPFAR-funded health facilities in East Africa: a cross-sectional survey with onsite verification.* Palliat Med, 2014. **28**(4): p. 293-301.
41. Merriman, A. and R. Harding. *Pain control in the African context: the Ugandan introduction of affordable morphine to relieve suffering at the end of life.* Philosophy, ethics, and humanities in medicine : PEHM, 2010. **5**: p. 10-10.
42. Khumalo, T. and V. Maasdorp. *The Island Hospice model of palliative care.* Ecancermedicalscience, 2016. **10**: p. 654-54.
43. Fund, U.N.C.s., *Assessment of the Need for Palliative Care for Children: Three Country Report: South Africa, Kenya and Zimbabwe*, S.R. Connor and C. Sisimayi, Editors. 2013.
44. Care, Z.M.o.H.a.C., *The National Palliative Care Policy*, T.D.o.E.D.C.N.-C.D. Unit, Editor. 2014, Zimbabwe Ministry of Health and Child Care: Harare, Zimbabwe.
45. African Palliative Care Association, *Review of the status of palliative care in ten Southern African countries.* 2016, African Palliative Care Association: Kampala, Uganda.
46. Worldwide Palliative Care Alliance, *Global Atlas of Palliative Care at the End of Life*, S.R. Connor and M. Bermedo, Editors. 2014, World Health Organization: London.
47. Gale, N.K., G. Heath, E. Cameron, et al. *Using the framework method for the analysis of qualitative data in multi-disciplinary health research.* BMC Medical Research Methodology, 2013. **13**(1): p. 117.
48. Nabudere, H., E. Obuku, and M. Lamorde. *Advancing palliative care in the Uganda health system: an evidence-based policy brief.* International journal of technology assessment in health care, 2014. **30**(6): p. 621-5.
49. Downing, J., M. Leng, and L. Grant. *Implementing a Palliative Care Nurse Leadership Fellowship Program in Uganda.* Oncology Nursing Forum, 2016. **43**(3): p. 395-98.

For peer review only - http://bmjopen.bmj.com/site/about/guidelines.xhtml

Appendix A: Palliative care reports undertaken in participating countries over the last 15 years

Country	Report	Key findings
Nigeria	Federal Ministry of Health: Treat the Pain (2012 – 2015):	• Around 177,000 people were estimated to have died in moderate or severe pain from HIV or cancer.<br>• The utilized narcotic medicines such as morphine was enough to treat only 266 people, representing 0.2% coverage of pain treatment
	Hospital Pain Free Initiative (2015 – date):	• Program to improve access to essential pain medicines to reduce needless suffering from cancer<br>• Pharmacist employed into Federal Ministry of Health specifically to coordinate the project<br>• 19.2kg of pulverized morphine imported, enough to treat about 3,000 patients.<br>• Strengthening the skills of health workers in pain assessment and management and equip them to provide high- quality pain treatment to their patients in line with World Health Organization (WHO) guidelines.<br>• Four teaching hospitals selected for pilot project (Based on their palliative care activity and utilization of oral morphine solution). Expanded to 15 by 2018
	National Comprehensive Cancer Network Harmonized Guidelines for Sub-Saharan Africa (2018 - 2019):	• Stakeholder workshops held leading to regional resources created as part of a collaborative efforts to combat rising cancer rates and unique care including palliative care
Uganda	Ministry of Health and National Drug clinical guidelines (2003):	• Nurses and Clinical officers are allowed to prescribe morphine for pain management
	Uganda Health Strategic plan, 2006–2011:	• Palliative Care is fully incorporated into the National Health Sector Strategic plan for the first time
	Evidence base for palliative care (2015)[48]	• A lack of policies and guidelines to support the development of palliative care was noted<br>• Plans to take forward palliative care development as part of the national health strategy
	Implementing a palliative care nurse leadership	• Trained palliative care nurses and clinical officers can effectively prescribe oral morphine

For peer review only

	fellowship program in Uganda (2016)[49]	
	Atlas of palliative care development (2017):	• Number of palliative care service outlets reported at 229<br>• Number of pediatric palliative care services reported at 2<br>• Has specialised training programmes for palliative care<br>• Palliative care services per million inhabitants reported at 5.87<br>• Has a Palliative Care National Association<br>• Oral morphine is produced locally
	National Cancer Control plan (2018):	• In the background based on an evidence brief for need for control strategy palliative care is recognised as part of the cancer control plan , and that it should be evidence based<br>• The Development of the NCCPs is underway, with a draft in place and palliative care is inclusive<br>• The NCCP addresses six key elements: (1) prevention, (2) early detection and screening, (3) diagnosis and treatment, (4) palliative care and (5) policy advocacy (6) research
	National eHealth strategy (2018):	• Baseline survey shows fragmented landscape of ICT pilot projects and numerous data and health information system (HIS) silos with significant barriers to the effective sharing of information between healthcare participants.<br>• Uganda adopts eHealth strategy to guide the use of ICT in supporting health sector transformation by addressing
Zimbabwe	WHO pilot study of palliative care in five African counties (2004): https://www.who.int/cancer/palliative/projectproposal/en/	• Estimated 1/60 people in Zimbabwe in need of palliative care<br>• Low level of palliative integration in the health system despite long history<br>• Training is a major strength<br>• Erratic pain relief
	African Palliative Care Association Policy and Gender Review in 10 countries (2010):	• Minimal coverage of palliative care<br>• No policies reviewed facilitated opioid availability<br>• Revealed gaps to be addressed to scale up integration of palliative care<br>• Recommended development of palliative care policy to guide actions and support for palliative care issues at national level

	National Palliative Care Situational Analysis commissioned by HOSPAZ (2012):	• Lack of knowledge and skills including listening and communication skills among care providers at all levels<br>• Need exists for palliative care training, bereavement counselling and home-based care<br>• Lack of knowledge / expertise in pain assessment and management using WHO analgesic ladder<br>• Unavailability and erratic supply of pain control medicines, especially strong opioids<br>• Children either not part of palliative care process or health providers have difficulty in handling their unique requirements (e.g. pain assessment and counselling)<br>• Poor integration of palliative care into public sector<br>• Lack of palliative care awareness and limited provision
	UNICEF and ICPCN Assessment of the Need for Palliative Care for Children: Three Country Report (2013):	• Number of children benefiting from palliative care services significantly lower than the estimated need (6% of specialised need)<br>• Lack of knowledge and adequate understanding of palliative care and children's palliative care among health professionals<br>• Lack of palliative care awareness by general public hence limited demand<br>• Poor pain management in children<br>• Morphine prescribing limited to medical doctors which is problematic
	African Palliative Care Association Morphine Survey (2015):	• Some progress toward integration of palliative care into health systems<br>• Advocacy for legal and policy changes recommended for nurse-prescribing of strong pain-relieving drugs, including opioids<br>• Opioid use in 2012 only 10.1kg instead of expected 177kg deduced from disease burden<br>• Recommended relevant knowledge on use of opioid analgesics to be integrated into medical and nursing school curricula
	Treat the Pain: A Country Snapshot (2016):	• Estimated 80% of cancer deaths (2012/2013) experience moderate to severe pain<br>• Average morphine consumption for 2011-2013 estimated 15.6kg, much lower than anticipated use<br>• Coverage of deaths in pain with treatment: 6%

For peer review only

Appendix B: Facilities involved in identification and recruitment of participants

Country	Facility name	Type of facility	Participants to recruit from facility
Nigeria	Lagos University Teaching Hospital	Tertiary care facility	Patients, caregivers, health professionals
	Sebeccly Cancer Care and Support Center	Private not-for- profit, community organisation	Patients, caregivers, health professionals
	National Ministry of Health, Nigeria	National government office	Policymakers
Uganda	Uganda Cancer institute (UCI)	Tertiary care medical facility	Patients, caregivers, health professionals
	Makerere Palliative Care Unit, Mulago Hospital	National referral hospital	Patients, caregivers, health professionals
	Kawempe Home Care	Private not-for- profit, community organisation	Patients, caregivers, health professionals
	Hospice Africa Uganda	Private not-for- profit, organisation with three facilities across Uganda	Patients, caregivers, health professionals
	Uganda Ministry of Health	National government office	Policymakers
Zimbabwe	Chitungwiza Hospital	Central hospital	Patients, caregivers, health professionals
	Parirenyatwa Group of Hospitals	Central hospital	Patients, caregivers, health professionals
	National Ministry of Health staff	National government office	Policymakers
	Island Hospice and Healthcare	Private not-for- profit, community organisation	Patients, caregivers, health professionals

For peer review only

BMJ Open

Understanding data and information needs for palliative cancer care to inform digital health intervention development in Nigeria, Uganda and Zimbabwe: protocol for a multi-country qualitative study

Journal:	*BMJ Open*
Manuscript ID	bmjopen-2019-032166.R1
Article Type:	Protocol
Date Submitted by the Author:	08-Oct-2019
Complete List of Authors:	Okunade, Kehinde ; University of Lagos College of Medicine, College of Medicine<br>Bashan Nkhoma, Kennedy; King's College London, Florence Nightingale Faculty of Nursing Midwifery and Palliative Care<br>Salako, Omolola ; Lagos State University Teaching Hospital, Department of Radiation Oncology<br>Akeju, David; University of Lagos, Department of Sociology<br>Ebenso, Bassey; University of Leeds, Nuffield Centre for International Health and Development;<br>Namisango, Eve; African Palliative Care Association<br>Soyannwo, Olaitan ; University College Hospital Ibadan<br>Namukwaya, Elizabeth; Makerere University College of Health Sciences, Palliative care unit, Department of Internal Medicine<br>Dandadzi, Adlight ; University of Zimbabwe College of Health Sciences, Clinical Trials Research Centre<br>Nabirye, Elizabeth; Makerere University College of Health Sciences, Palliative care unit, Department of Internal Medicine<br>Mupaza, Lovemore; Island Hospice and Healthcare<br>Luyirika, Emmanuel; African Palliative Care Association<br>Ddungu, Henry; Uganda Cancer Institute<br>Chirenje, Z. Mike ; University of Zimbabwe College of Health Sciences<br>Bennett, Michael; University of Leeds, Academic Unit of Palliative Care, Leeds Institute of Health Sciences<br>Harding, Richard; King's College London, Florence Nightingale Faculty of Nursing Midwifery and Palliative Care<br>Allsop, Matthew ; University of Leeds, Academic Unit of Palliative Care, Leeds Institute of Health Sciences
**Primary Subject Heading**:	Palliative care
Secondary Subject Heading:	Global health, Health services research, Health informatics, Qualitative research
Keywords:	PALLIATIVE CARE, ONCOLOGY, Information technology < BIOTECHNOLOGY & BIOINFORMATICS, International health services < HEALTH SERVICES ADMINISTRATION & MANAGEMENT, QUALITATIVE RESEARCH, PUBLIC HEALTH

For peer review only - http://bmjopen.bmj.com/site/about/guidelines.xhtml

Understanding data and information needs for palliative cancer care to inform digital health intervention development in Nigeria, Uganda and Zimbabwe: protocol for a multi-country qualitative study

Author list: Kehinde Okunade[1], Kennedy Bashan Nkhoma[2], Omolola Salako[3], David Akeju[4], Bassey Ebenso[5], Eve Namisango[6], Olaitan Soyannwo[7], Elizabeth Namukwaya[8], Adlight Dandadzi[9], Elizabeth Nabirye[8], Lovemore Mupaza[10], Emmanuel Luyirika[6], Henry Ddungu[11], Mike Chirenje[9], Michael I Bennett[5], Richard Harding[2], Matthew J Allsop[5]*.

Author affiliation:

[1] College of Medicine, University of Lagos, University of Lagos, Lagos, Nigeria

[2] Florence Nightingale Faculty of Nursing Midwifery and Palliative Care, Cicely Saunders Institute, Kings College London, UK

[3] Department of Radiation Oncology, Lagos University Teaching Hospital, Lagos State, Nigeria

[4] Department of Sociology, University of Lagos, University of Lagos, Lagos, Nigeria

[5] Academic Unit of Palliative Care, Leeds Institute of Health Sciences, University of Leeds, Leeds, UK

[6] African Palliative Care Association, Kampala, Uganda

[7] Centre for Palliative Care, University College Hospital, Ibadan, Nigeria

[8] Department of Internal Medicine, Makerere University, Kampala, Uganda

[9] Clinical Trials Research Centre, College of Health Sciences, University of Zimbabwe, Harare, Zimbabwe

[10] Island Hospice and Healthcare, Harare, Zimbabwe

[11] Uganda Cancer Institute, Kampala, Uganda

**Corresponding author:* Dr Matthew Allsop, Academic Unit of Palliative Care, Leeds Institute of Health Sciences, University of Leeds, Worsley Building Clarendon Way, LS2 9NL, Leeds, UK. Email: m.j.allsop@leeds.ac.uk. Phone: +44(0)113 343 4185.

Word count: 2,994 words

For peer review only - http://bmjopen.bmj.com/site/about/guidelines.xhtml

For peer review only

ABSTRACT

Introduction: Palliative care is a clinically and cost-effective component of cancer services in SSA. Despite the significant need for palliative cancer care in SSA, coverage remains inadequate. The exploration of digital health approaches could support increases in the quality and reach of palliative cancer care services in SSA. However, there is currently a lack of any theoretical underpinning or data to understand stakeholder drivers for digital health components in this context. This project addresses this gap through engaging with key stakeholders to determine data and information needs that could be supported through digital health interventions.

Methods and analysis: This is a multi-country, cross-sectional, qualitative study conducted in Nigeria, Uganda and Zimbabwe. In-depth interviews will be conducted with patients with advanced cancer (n = 20), caregivers (n = 15), health professionals (n = 20) and policy makers (n = 10) in each of the three participating countries. Data from a total of 195 interviews will transcribed verbatim and translated into English before being imported into NVivo software for deductive framework analysis. The analysis will seek to understand the acceptability and define mechanisms of patient-level data capture and usage via digital technologies.

Ethics and dissemination: Ethics approvals have been obtained from the Institutional Review Boards of University of Leeds (Ref: MREC 18-032), Research Council of Zimbabwe (Ref: 03507), Medical Research Council of Zimbabwe (Ref: MRCZ/A/2421), Uganda Cancer Institute (Ref: 19-2018), Uganda National Council of Science and Technology (Ref: HS325ES), and College of Medicine University of Lagos (Ref: HREC/15/04/2015). The project seeks to determine optimal mechanisms for the design and development of subsequent digital health interventions to support the development, access and delivery of palliative cancer care in SSA. Dissemination of these findings will occur through newsletters and press-releases, conference presentations, peer-reviewed journals and social media.

Trial registration number: ISRCTN15727711

Key words: palliative care, oncology, information technology, international health services, qualitative research, public health

For peer review only

ARTICLE SUMMARY

- This study is the first to identify preferences of stakeholders involved in palliative cancer care provision in sub-Saharan Africa to inform digital health approaches
- All aspects of the proposed study have been coproduced with experts in palliative care delivery and research in Nigeria, Uganda and Zimbabwe
- This multi-country study will generate a logic model to target digital health approaches for palliative care that could have relevance across the sub-Saharan Africa region
- This study restricts its focus to palliative cancer care so its relevance to other palliative conditions may be limited
- The findings may not be reproducible beyond the three participating countries.

For peer review only

For peer review only - http://bmjopen.bmj.com/site/about/guidelines.xhtml

INTRODUCTION

Due to late-stage clinical presentation, limited funding and restricted access to curative therapies, about 80% of cancers on the continent are incurable at the time of detection and diagnosis.[1] In 2018, there were over 770,000 new cancer cases and 514,000 cancer-related deaths across Eastern, Middle, Southern and Western Africa.[2] These figures are projected to continue to rise (up to 1.28 million new cases and 970,000 deaths) by 2030, with subsequent international and regional political declarations constituting a new global non-communicable disease agenda.[3] Increasing incidence is attributed to factors that include ageing, high residual burden of infectious agents (HIV/AIDS, human papillomavirus, hepatitis B virus) and lifestyle factors in sub-Saharan Africa (SSA).[4] Our analysis reveals that by 2060, an estimated 16 million people with cancer will die annually with serious health-related suffering, a 109% increase between 2016 and 2060, with the fastest rise occurring in low-income countries (400% increase).[5]

Palliative care (PC)—the prevention and relief of physical, emotional, social, or spiritual suffering associated with any chronic or life-threatening illness, from the point of diagnosis—is a vital and fundamental component of the basic and essential services within Universal Health Coverage (UHC).[6] It is also a realistic response to support equitable, accessible and cost-effective interventions for cancer care in SSA. Independent of cancer prevention and treatment efforts in the region, PC remains a critical and essential component of care, with proven effectiveness and cost effectiveness.[7, 8] There have been enormous strides made in the development of PC services in the SSA region[9, 10] but there remains a need for significant expansion of provision to meet demand. Current provision of PC services is limited to 24 of 48 countries, up from only five in 2004, with only less than 5% of people who need PC being able to access services in the region.[10]

A major challenge to developing palliative cancer care across the African region is the lack of local evidence to ensure practice is evidence-based and replicable and reflects the needs of the population served.[6] Evidence to date has revealed that advanced cancer patients in SSA have a high burden of physical and psychological symptoms, would prefer to have full information and better communication around their needs and care options, experience spiritual distress, their family caregivers face compounded poverty and psychological distress.[11-18] It is essential to create channels for gathering patient-level data as an indicator of quality as well as to inform clinical practice and audit. Furthermore, understanding how emerging services are supporting patients with advanced cancer through assessing experiences and outcomes is a priority for PC development in the region. [19] This can be achieved through the use of validated, context-specific tools for measuring outcomes

For peer review only - http://bmjopen.bmj.com/site/about/guidelines.xhtml

for PC patients and their caregivers in SSA.[19, 20] We have therefore developed a valid patient-reported outcome measure (PROM) for advanced disease in SSA to capture the core concerns of patients and families.[21-23] Capturing these data can enable development and adaptation of services to ensure they can respond to the specific needs of patients with cancer.

This protocol describes a study to explore how technology-based approaches could capture patient-level data from patients with advanced cancer that has utility across the health system. Previous work by our team has highlighted the potential of digital health to facilitate the collection, sharing and use of patient-level data. For example, we know that mobile phones are frequently used in multiple ways as part of PC service provision in the African region and that development of approaches that capitalise on mobile phones is a high priority for providers.[24] In SSA, mobile phone services are available to a larger portion of the population than many basic services (such as sanitation and financial services). Approaches using digital health can benefit from the widespread access and low cost of mobile phone devices in the region and have shown improved chronic disease management;[24] patient behaviour change and health systems strengthening;[25] reduced costs of patient monitoring; improved adherence; and better communication. These benefits are greatest in rural areas[26]. Furthermore, such patient to provider telemedicine has recently been recommended by the World Health Organization (WHO) as an approach that can support health systems strengthening.[27]

In recent years, there has been exploratory research and development of digital health approaches in PC services in SSA.[28-30] However, this project seeks to address the lack of a theoretical underpinning to interventions using digital health components in this context. Our project will undertake engagement with key stakeholders (patient, caregivers, health professionals and policymakers) across the health system to define the optimal mechanisms through which patient-level data, captured via digital health approaches, can be integrated into palliative cancer care delivery and improvement.

Research objectives and questions

The study aims to answer the question, what are the optimal mechanisms through which patient-level data, captured via digital health, can be used in the development and delivery of palliative cancer care in sub-Saharan Africa?

The specific objectives of the project are to:

1. Establish a consortium of academic researchers (from the UK, Nigeria, Uganda, and Zimbabwe), service user advocates, non-governmental organisations, palliative care

For peer review only - http://bmjopen.bmj.com/site/about/guidelines.xhtml

providers, policymakers and digital health development and implementation experts, to catalyse digital health research and generate evidence that can guide palliative cancer care development across sub-Saharan Africa.

2. Understand the acceptability and optimal implementation of patient-level data collection (e.g. patient-reported outcome measures and patient-reported experience measures) using digital health approaches in Uganda, Nigeria and Zimbabwe through patient and caregiver engagement.
3. Determine information needs and pathways for leveraging evidence generated from digital health approaches in service development in Uganda, Nigeria and Zimbabwe through health professional and service manager engagement.
4. Determine information needs and pathways for leveraging evidence generated from digital health approaches in policymaking in Uganda, Nigeria and Zimbabwe through policymaker engagement.
5. Define the mechanisms for implementation of digital health approaches to support development of palliative cancer care in sub-Saharan Africa.
6. Develop a theoretically-informed logic model for implementing digital health approaches to improve palliative care in sub-Saharan Africa.

METHODS AND ANALYSIS

Through partnership with the African Palliative Care Association (APCA), the University of Leeds have assessed the use and priorities of digital health approaches in PC services in the African region.[28] Furthermore, Kings College London, working with APCA, have been pioneering patient-level data collection in PC in SSA and developing Patient Reported Outcome Measures (PROMS);[19, 20] simple checklists of symptoms and concerns, that are widely adopted and enable staff, patients and families to identify main concerns and prevent suffering, maintain people at home, support families, and optimise function. PROMS for people with serious incurable illness can improve care and patient wellbeing. This project will enable integration of these research initiatives, determining the architecture of digital technologies to facilitate uptake and utilisation of evidence-based approaches such as PROMS. This will take forward the science of digital health in this neglected field, enabling a logic model to be developed for subsequent evaluation and implementation.

Palliative care development in participating countries

The current development of PC for each participating country is summarised in Table 1.

For peer review only - http://bmjopen.bmj.com/site/about/guidelines.xhtml

Table 1: Summary of palliative care development in Nigeria, Uganda and Zimbabwe

Country	Summary of development
Nigeria	In Nigeria – the most populous country in SSA – palliative care (PC) is disparately spread in centres across the country. Historically, PC development in western Africa has been secondary to developments in the eastern and southern parts of the continent, and this continues to be the case. This is due mainly to the relatively low HIV prevalence, which meant that the region did not qualify to receive funding from the US President's Emergency Plan for AIDS Relief (PEPFAR) in the early 2000s.[31] Widespread interest in palliative care development in Nigeria emanated from the pain and suffering witnessed by concerned health professionals in patients with advanced cancer. Early efforts by the Hospice Nigeria team included an advocacy visit by Anne Merriman (the founder of Hospice Africa Uganda in 1993) which yielded little result as there was no opioid analgesics in the country to manage the associated cancer pain.[32] The current movement that has resulted in establishment of holistic palliative care services across Nigeria commenced in 1996 with the Ibadan "Cancer pain group".[33] Concerted advocacy and other activities of the group facilitated importation of opioid analgesics by the Federal Ministry of Health for management of severe pain by 2001 and morphine powder for oral morphine preparation by 2005.<br>The first PC team was founded in 2003 at Ibadan, providing both hospital- and home-based services,[31] and the Hospice and PC Association of Nigeria was created in 2007. Members, most of whom received palliative care initiator's training at Hospice Africa, Uganda function as individuals or teams across the six geopolitical areas of the country. Home-based PC continues as a very important form of PC delivery, with evidence of its benefits to patients and their families.[34] Some aspects of palliative care are now included in the national guidelines for HIV and AIDS treatment and care in adolescents and adults (2012) as well as the national cancer control plan of the FMOH (2018-2022)<br>Current barriers to PC development include lack of government guidelines, poor knowledge about PC importance at all levels – policy makers, public, health professionals, lack of inclusion of palliative care in curricula of health professionals and in the national health budget and national health Insurance (NHIS), poor availability and accessibility of strong opioid analgesics for cancer pain management. However, since 2012, the 'Treat Pain project' and "Pain Free

For peer review only - http://bmjopen.bmj.com/site/about/guidelines.xhtml

	Hospital Initiative" of the Federal Ministry of Health, Global Access to Pain Relief Initiative (GAPRI) and American cancer Society have improved the opioid situation.[35, 36] More can still be achieved through the training of more health professionals and change of attitude, increased funding, and increased public awareness of the services.[34] There has been some early context setting work suggesting willingness to explore the application of technology within PC services, such as the use of telemedicine.[37]
Uganda	The Atlas of Palliative Care Development in Africa ranked Uganda highly in terms of palliative service development and its integration into the health system.[38] Prior to this, The Economist intelligence unit quality of death index[39] which is a "measure of the quality of palliative care provided to adults in over 80 countries", ranked Uganda as 35th in the world. This index gives a general impression of the quality of life at the end of life and recognises continued development of PC services in Uganda. In terms for service delivery, Uganda has over 229 palliative care service outlets, for a population of 40 million people. These include hospices, home-based care and health facility-based outlets. The level of pediatric palliative care service development remains poor, with two pediatric palliative care service centres despite a large population of children and young people.[1]<br>In terms of the wider health system, Uganda has been progressing the presence and development of palliative care services. Uganda is building a critical mass for palliative care professionals with palliative care integrated into the medical and nursing and is a hub for training and education in palliative care, with diploma, certificate and degree courses in palliative care provided by universities, specialised palliative care institutions such as Mildmay Uganda, and international organisations such as the African Palliative Care Association. In terms of health information systems, there are two national indicators of palliative care into the national electronic health information system; patients presenting with pain and those receiving morphine for pain management. With this advancement, to some extent, performance of palliative care can be monitored at the national level.<br>Access to pain medication is a crucial component of palliative care delivery in Uganda. The availability of the full analgesic ladder and palliative medicines remains poor,[40] compromising care providers' ability to support management of symptoms and alleviate suffering associated with pain. However, Uganda has introduced prescribing across cadres other than doctors, such as palliative care

For peer review only - http://bmjopen.bmj.com/site/about/guidelines.xhtml

	nurse prescribers. Furthermore, the rolling out of local production of morphine within Hospice Africa Uganda led to significant reductions in costs associated with morphine procurement.[41] This key opioid for moderate to severe pain is now provided free of charge and is supplied through the national supply chain mechanisms of the country. Although oral morphine is free, access still remains a challenge because of several factors such frequent stock outs, reluctance to prescribe morphine for fear of addictions as well as limited access to this medicine at primary health care level.[29]<br>Palliative care is included in the national health strategic plan, and in the HIV care guidelines. A standalone palliative care policy was drafted in 2017 and is pending cabinet approval. It is envisioned that a standalone policy will be a strong pillar in giving a strategic direction to service development and evaluation to provide a comprehensive framework for the integration of palliative care services into the health care system. It will also be used as tool to advance financing for palliative care advocacy.
Zimbabwe	In 2014, a WHO report identified 1 in 60 Zimbabweans need palliative care,[42] alongside an International Children's Palliative Care Network report identifying a significant need among children across multiple provinces in Zimbabwe.[43] Despite the continuing high level of palliative care need, Zimbabwe was the first country in sub-Saharan Africa to have a hospice, founded in 1979. In terms of development of palliative care across the country, by 1997 there were 17 regional branches that had been formed throughout the country with 13 organisations providing palliative care by 2004.[44] The disease focus of palliative care in Zimbabwe was initially cancer, although the growth of the disease burden due to HIV and AIDS led to widening of provision to include those living with HIV and AIDS and other chronic illnesses. Notable initiatives have facilitated palliative care services provision in the country. In 1992, the Ministry of Health and Child Care formed the Prevention and Control of Cancer Committee in Zimbabwe that comprised relevant stakeholders and professionals. The committee oversaw the development of a ten-year National Cancer Control Programme Plan for Zimbabwe (1994 – 2004), with the overall aim to formulate, plan and implement a coordinated and cost-effective programme for the prevention and control of cancer in Zimbabwe. This led to the establishment of palliative care training across eight provinces and two cities of Harare and Bulawayo. The African Palliative Care

For peer review only - http://bmjopen.bmj.com/site/about/guidelines.xhtml

	Association (APCA) in 2010 reviewed national policy documents and implementation guidelines from ten Southern African countries, including Zimbabwe.[45] For Zimbabwe, whilst palliative care was highlighted as a priority area across documents, issues around minimal coverage of palliative care was noted alongside an absence of detail relating to opioid availability. In more recent years the Worldwide Hospice Palliative Care Association (WHPCA) global update of mapping levels of palliative care provision in 2014, placed Zimbabwe in category 4a.[46] Category 4a suggests a country as hospice-palliative care services at a stage of preliminary integration into mainstream service provision. This category suggests the development of a critical mass of palliative care activism in a number of locations, a variety of palliative care providers and types of services, awareness of palliative care on the part of health professionals and local communities, the availability of morphine and some other strong pain-relieving medicines, limited impact of palliative care upon policy, the provision of a substantial number of training and education initiatives by a range of organisations, and interest in the concept of a national palliative care association.

In recent years, policy reports of PC from participating study countries have commonly identified priority focus areas of improving access to pain medications, improving awareness by health professionals of the value and role of PC in supporting patients, and integration of PC with the existing public health system. A summary of policy documents and their key findings for each country is provided in Appendix A.

For peer review only

Study design

This study will adopt a multi-country, cross-sectional, exploratory study using qualitative methods. In-depth interviews with patients, caregivers, health professionals and policy makers will be used to understand the acceptability and define mechanisms of patient-level data capture and usage. The interpretation of the findings will define optimal mechanisms through which patient-level data, captured via digital health, can be used in the development, delivery and improvement of palliative cancer care in sub-Saharan Africa. Alongside the planned research activities, a consortium focused on digital technology for palliative cancer care will be formed. This will include researchers from SSA and the UK, alongside key stakeholders in each of the participating countries (i.e. relevant policy representatives from ministries of health, civil society, patient advocates, and digital health specialists) to understand: i) current digital health development in Uganda, Nigeria and Zimbabwe; ii) opportunities for capacity development around digital health in palliative cancer care, and; iii) routes to uptake and translation of findings from planned research activities.

Study participants

The study participants will be adults living with advanced cancer, their caregivers, their health professionals, and policymakers with a focus on cancer, non-communicable diseases and/or technology. Patients will include adults with advanced cancer receiving palliative care. Caregivers will include those supporting palliative cancer patients receiving care from recruiting facilities. Health professionals will be drawn from the clinical teams associated with study partner institutes alongside related services delivered in the locality. Policymakers will comprise district or national level policymakers working within cancer, non-communicable diseases, or digital health. Table 2 outlines the inclusion / exclusion criteria, sampling characteristics and sample size for the four stakeholder groups.

For peer review only - http://bmjopen.bmj.com/site/about/guidelines.xhtml

Table 2: Population and sampling criteria for the four stakeholder groups

Participant group	Eligibility criteria	Sampling criteria	Target number to recruit in each participating country
**Patient**	***Inclusion criteria:***<br>• Patients with advanced cancer (defined as those with metastatic cancer (where, if possible, determined through histological, cytological or radiological evidence) and/or those receiving anti-cancer therapy with palliative intent) receiving palliative care<br>• Aged ≥ 18 years<br>***Exclusion criteria:***<br>• Patients with significant cognitive impairment that prevents informed consent<br>• Inadequate physical and mental health of a patient prior to recruiting (as deemed by the identifying clinician)<br>• Lack of a shared language between researcher and respondent<br>• Below 18 years of age	• Age<br>• Sex<br>• Cancer type<br>• Location at time of interview (community-based or ward-based)	• 20 purposely selected patients
**Caregiver**	***Inclusion criteria:***<br>• A primary caregiver/family caregiver of a patient with advanced cancer who is at least 18 years of age<br>• Primary caregiver confirmed by the patient, including those who provide unpaid, informal provision of one or more physical, social, practical and emotional tasks. In terms of their relationship to the	• Sex<br>• Age<br>• Patient involvement in study (ensuring representation of those where the patient whom they care for has participated,	• 15 purposely selected caregivers

For peer review only

For peer review only - http://bmjopen.bmj.com/site/about/guidelines.xhtml

	patient, they may be a friend, partner, ex-partner, sibling, parent, child or other blood or non-blood relative<br>• Both caregivers of participating and non-participating patients will be recruited into the study<br>***Exclusion criteria:***<br>• Below 18 years of age<br>• Inadequate physical and mental health of a patient prior to recruiting (as deemed by the identifying clinician)	and those where the patient has not participated).	
**Health professional**	***Inclusion criteria:***<br>• Working with palliative care patients<br>• At least six months working experience at recognised palliative care facilities	• Role (doctor, clinical officer, nurse, social worker, psychologist, pharmacist)<br>• Typical work setting (i.e. community-based, ward-based)	• 20 purposely selected health professionals
**Policymaker**	***Inclusion criteria:***<br>• Representative from government ministry or national association responsible for oversight and development of healthcare in participating country	• Working at different levels of the health system (district, national)<br>• Policy remit of their post (cancer, non-communicable diseases, digital health)	• Up to 10 purposely selected policymakers

For peer review only - http://bmjopen.bmj.com/site/about/guidelines.xhtml

For peer review only

Data collection and management

Patients

Clinical staff at recruiting facilities will be responsible for identifying participants. These staff will be asked to verify that patients are aware of their palliative care diagnosis. This will be through review of clinical records and discussion with the participant's health professionals by the research assistant. Patients who are deemed ethically inappropriate by members of the clinical team, for example where death is imminent, will not be approached. Participating patients will only be asked to participate in one face-to-face interview. The location of the interview will be decided by the patient or caregiver (i.e. meeting either at their home, following a clinic appointment at a health facility, on the ward, or at a neutral location, dependent on the patient's preference and the patient's clinical management at the time of the interview). Independent of the location chosen, a quiet and private room will be recommended to the patient for the interview. Arrangements will be made for such space at clinics and hospices ahead of interviews. A topic guide will be used to direct semi-structured interviews with patients. The topic guide will explore current interaction with and access to PC services, their use of technology, the acceptability of using digital technology approaches to support interaction with health services and data collection, the clinical response anticipated from health services (with and without facilitation by digital health interventions) including perceptions of effective responses, and alternative approaches to patient-level data collection without mobile phones.

Caregivers

Caregivers of patients with advanced cancer will be identified and approached to participate by clinical staff at recruiting facilities. Caregivers will only be required to participate in one face-to-face interview. A topic guide will be used to direct semi-structured interviews with caregivers. The themes addressed in the topic guide will align with the patient topic guide.

Healthcare professionals

Health professionals will be identified by clinical leads in each of the three countries. Through existing networks of PC providers in each of the countries, the clinical lead will approach health professionals to participate in the study. A topic guide will be used during health professional interviews. The topic guide aligns with stages of the data-use conceptual framework[15]: data demand (e.g. current availability, use and quality of data for clinical decision making), data collection (e.g. feasibility of digital technology approaches to patient-level data collection), data availability (e.g. clinical triggers in management of patients with advanced cancer, capacity to respond to information, information needs to inform patient care), and data utilization (e.g. sharing and accessing data via digital health

For peer review only - http://bmjopen.bmj.com/site/about/guidelines.xhtml

approaches, data reporting priorities to regional and national health authorities). Causal elements linked to organisational, technical and behavioural factors influencing data use will be explored for each stage.

Policymakers

Policymakers will be identified and approached by the African Palliative Care Association alongside academic and clinical teams in Nigeria, Uganda and Zimbabwe. A topic guide for key informant interviews will address access to and use of evidence to inform decision making, seek comment on findings from a desktop review of existing policy on digital health to be conducted prior to key informant interviews, preferred mode and presentation of data, frequency of data reporting needed to inform decision-making on financing of PC services and on accelerating universal health coverage.

Interviews across all recruitment sites in Uganda, Nigeria and Zimbabwe (outlined in Supplementary Appendix B) will be undertaken by research assistants, supervised by the academic partners in Nigeria (KO), Uganda (LN) and Zimbabwe (MC). All interviews will be audio recorded. Data security will be ensured through use of password-protected file sharing using the Microsoft OneDrive platform. Separate folders will be created for each country, with oversight from the lead institution, the University of Leeds. Only research team members will have access to the folder. All members of the project team will sign a data sharing agreement outlining explicit guidance regarding handling and management of research data that takes account of both the funders and national research council guidelines. On completion of this study, all electronic data on the OneDrive folder will be moved to an electronic archive for five years prior to being permanently destroyed. De-identified research data deemed suitable for sharing will be hosted by Research Data Leeds, the institutional research data repository for the University of Leeds. All study participants will be assigned an identification code, which will be delinked from their identity at data entry point.

Reflectivity

The research team comprises experts in digital health intervention development (MA, BE), qualitative research (DA, AD, EN), global PC provision (KBN, EN, EL, RH), monitoring and evaluation (LM), health services research in SSA (MC) alongside consultants in palliative medicine (EN, HD, OS, MB) and oncologists (KO, OS). None of these researchers have any relationship with the patients or caregivers who will be approached to participate. However, a small number of the healthcare professionals or policymaker participants may be known contacts.

For peer review only - http://bmjopen.bmj.com/site/about/guidelines.xhtml

Patient and public involvement

The rationale for this work arose from priorities identified through surveying a key stakeholder group, healthcare providers delivering palliative care across sub-Saharan Africa.[24] Patients and caregivers were not involved in the design of the study. However, alongside research activities, the team will develop a consortium which will include patient advocates. This forum will be used to explore routes for communicating study findings to patient groups and help to establish potential routes for identifying patient and caregivers, or advocates, in subsequent projects.

Study dates

November 2018 – December 2019. Policymaker engagement will begin November 2018, with data collection commencing March 2019.

Data Analysis

Interviews will be transcribed verbatim and translated into English before being imported into NVivo software for deductive framework analysis.[47] An initial charting of pseudonymised transcripts (KN) will be developed through line-by-line coding. This initial code will then be applied to a random set of transcripts (n=3) in each stakeholder group by three further members of the research team (Nigeria, Uganda, Zimbabwe), and the final framework agreed through discussion with the wider team. Once inconsistencies are resolved, the joint coding frame will be used to code all interviews across the participant group. Comparative analysis in the framework will enable us to identify common themes as well as country-specific and stakeholder group divergences. A model of the coding frame will be developed, and each theme and subtheme given a definition to ensure internal consistency of each code. Illustrative codes will be reported for each theme, with the study ID code to demonstrate reporting from across the sample breadth. In the final project meeting we will hold a Theory of Change workshop to model the planned digital health pathway within its context, detailing processes, stakeholder roles, outcomes and intended impact.

Ethical considerations

Ethics approvals have been obtained from the Institutional Review Boards of University of Leeds (Ref: MREC 18-032), Research Council of Zimbabwe (Ref: 03507), Medical Research Council of Zimbabwe (Ref: MRCZ/A/2421), Uganda Cancer Institute (Ref: 19-2018), Uganda National Council of Science and Technology (Ref: HS325ES), and College of Medicine University of Lagos (Ref: HREC/15/04/2015). Ethical review undertaken by all project investigators ensured standard processes (dignity, autonomy, informed consent, confidentiality, anonymity, ability to adhere to protocol) and data security were

For peer review only - http://bmjopen.bmj.com/site/about/guidelines.xhtml

considered in the protocol development. The project will be aligned with the MRC good research practice guidelines and H3Africa framework for conducting ethically responsible biomedical research. With reference to research on palliative care populations, a patient's condition should not preclude them from participation. Instead, additional strategies of harm minimisation have been developed collaboratively by the research team. Academic leads and co-investigators will support protocol adaptation for local cultural appropriateness (e.g. modifying recruitment accounting for cancer-related stigma). Researchers will routinely contact clinical teams to check the health status of a patient prior to contact for research activities. In terms of data governance, project documentation and de-identified data for joint analysis will be shared via a secure IT infrastructure hosted by the University of Leeds. Voluntary and informed participation, confidentiality and safety of participants will constitute key principles of researcher–respondent interaction. Written consent or a thumb print will be obtained from patients, caregivers, health care professionals and policymakers prior to their enrolment in the study.

DISSEMINATION

We will provide an understanding of the mechanisms by which digital health approaches can facilitate evidence generation and use, ensuring optimal implementation with clear pathways for integrating captured data into existing health system functions. On completion of planned research activities, we will have:

1. Instigated an active, interdisciplinary consortium focused on technology-based approaches to developing palliative cancer care in SSA;
2. Defined mechanisms for optimal implementation of digital health interventions to support PC service development in SSA;
3. Provided a list of factors to target with digital health approaches with accompanying programme theory;
4. Obtained essential preliminary data needed to inform future research in digital health technology development for PC in SSA;
5. Developed a logic model for implementation of digital health to improve advanced cancer care in SSA.
6. Strengthened capacity for research, innovation and knowledge exchange in partner institutions and identified future capacity building needs.

Undertaking primary research with patients, caregivers, health professionals and policymakers is essential to guide digital health approaches for palliative cancer care in SSA. A number of activities will

For peer review only - http://bmjopen.bmj.com/site/about/guidelines.xhtml

be undertaking to support dissemination of project findings. These will include: i) developing newsletters and press-releases to communicate key project findings to the general public; ii) developing a dedicated website for the study where results will be publicly accessible by national and international policymakers, practitioners and academics; iii) delivering presentations at local and national conferences in participating countries, alongside presentation at international conferences; iv) publishing articles in peer-reviewed journals; and v) social media through research team member and institutional accounts. Participants will be anonymised in any dissemination activities. Only pseudonymised, non-identifiable characteristics and quotes will be used in dissemination.

CONCLUSION

This paper reports the protocol for a cross-sectional study with qualitative methods aimed at understanding the optimal mechanisms through which patient-level data, captured via digital health, can be used in the development, access and delivery of quality palliative cancer care in Uganda, Nigeria and Zimbabwe. The findings of the study will be device agnostic, providing a theoretical framework that can be used to inform a wide range of digital health intervention development and implementation. Without this research there is a risk of digital health intervention development for PC occurring in silos across SSA that do not take account of the multiple uses and value of data for stakeholders across the wider health system. Mapping information and data needs across PC services will also create multiple opportunities for research. This includes subsequent piloting and evaluation of digital health interventions and validation of the data they capture, development of patient-focused digital health interventions such as information provision and self-management support, and exploration of the influence of factors such as gender, intersectionality, disability and cancer type on utilisation and engagement with digital health approaches. Subsequent development of digital health approaches for PC in SSA, gathering patient-level data and facilitating patient-provider communication, could lead to multiple benefits for patients and caregivers (reduced costs associated with time and travel to facilities, extend coverage and reach of services such as rural areas with mobile connectivity), health professionals (ability to identify and respond to specific and rising demand from patients with cancer), and policymakers (receiving appropriate and timely data to inform service planning, guide integration of PC with wider healthcare delivery, and contribute to strengthening of national digital health systems). There is an added imperative to understand how best to utilise digital health technologies for those receiving and providing care for advanced disease in SSA. There is currently a lack of evidence on the preferences of patients with advanced disease, their caregivers and their health professionals. Understanding these preferences is crucial to inform digital health

For peer review only

For peer review only - http://bmjopen.bmj.com/site/about/guidelines.xhtml

intervention development, such as modelling interventions around the preferences of the timing and delivery of digital health approaches in PC.

PROTOCOL AND REGISTRATION

This study protocol is registered on the ISRCTN website (ISRCTN15727711).

ACKNOWLEDGEMENTS

We would like to thank Olasupo Oyedepo, Director of the African Alliance of Digital Health Networks and Project Director at ICT4HEALTH Project for guidance on the development and implementation of this project.

CONTRIBUTORS

MJA conceived the study; MJA, BE, RH, EN (Namisango), EN (Namukwaya) and MC contributed to the development of the study design and final protocols for sample selection, observations and interviews; KO, MJA, OS and EN (Namisango) developed a draft of the manuscript; All authors (MJA, KO, KBN, OS, DA, BE, EN, OS, EN, AD, EN, LM, EL, HD, MC, MIB, RH) contributed to writing the manuscript.

FUNDING

This work was supported by the Medical Research Council (grant number MR/S014535/1) and Research England Quality-related Global Challenges Research Fund (QR GCRF) through the University of Leeds.

COMPETING INTERESTS

None declared.

ETHICS

Ethics approvals have been obtained from the Institutional Review Boards of University of Leeds (Ref: MREC 18-032), Research Council of Zimbabwe (Ref: 03507), Medical Research Council of Zimbabwe

For peer review only - http://bmjopen.bmj.com/site/about/guidelines.xhtml

(Ref: MRCZ/A/2421), Uganda Cancer Institute (Ref: 19-2018), Uganda National Council of Science and Technology (Ref: HS325ES), and College of Medicine University of Lagos (Ref: HREC/15/04/2015).

DATA SHARING STATEMENT

No additional data available.

PROVENANCE AND PEER REVIEW

Not commissioned; externally peer reviewed.

For peer review only

REFERENCES

1. Ferlay, J., I. Soerjomataram, R. Dikshit, et al. *Cancer incidence and mortality worldwide: sources, methods and major patterns in GLOBOCAN 2012.* International journal of cancer, 2015. **136**(5): p. E359-86.
2. International Agenct for Research on Cancer, *Globocan: All cancers*. 2018, International Agenct for Research on Cancer: France.
3. Powell, R.A., Z. Ali, E. Luyirika, et al. *Out of the shadows: non-communicable diseases and palliative care in Africa.* BMJ Supportive & Palliative Care, 2015.
4. Jemal, A., P. Vineis, F. Bray, et al., *The Cancer Atlas*. 2016, Atlanta, Georgia: American Cancer Society.
5. Sleeman, K., M. Brito, S. Etkind, et al. *The escalating global burden of serious health-related suffering: projections to 2060 by world regions, age groups and health conditions. .* Lancet Global Health, 2019. **In Press**.
6. Knaul, F.M., P.E. Farmer, E.L. Krakauer, et al. *Alleviating the access abyss in palliative care and pain relief—an imperative of universal health coverage: the Lancet Commission report.* The Lancet, 2018. **391**(10128): p. 1391-454.
7. Gomes, B., N. Calanzani, V. Curiale, et al. *Effectiveness and cost-effectiveness of home palliative care services for adults with advanced illness and their caregivers.* Cochrane Database of Systematic Reviews, 2013(6).
8. Smith, S., A. Brick, S. O'Hara, et al. *Evidence on the cost and cost-effectiveness of palliative care: a literature review.* Palliat Med, 2014. **28**(2): p. 130-50.
9. Fraser, B.A., R.A. Powell, F.N. Mwangi-Powell, et al. *Palliative Care Development in Africa: Lessons From Uganda and Kenya.* Journal of Global Oncology, 2017(4): p. 1-10.
10. Downing, J., L. Grant, M. Leng, et al. *Understanding Models of Palliative Care Delivery in Sub-Saharan Africa: Learning From Programs in Kenya and Malawi.* Journal of Pain and Symptom Management, 2015. **50**(3): p. 362-70.
11. Beynon, T., E. Radcliffe, F. Child, et al. *What are the supportive and palliative care needs of patients with Cutaneous T cell Lymphoma (CTCL) and their caregivers? A systematic review of the evidence.* The British journal of dermatology, 2013.
12. Harding, R., L. Selman, G. Agupio, et al. *The prevalence and burden of symptoms amongst cancer patients attending palliative care in two African countries.* European journal of cancer, 2011. **47**(1): p. 51-6.
13. Selman, L., I.J. Higginson, G. Agupio, et al. *Meeting information needs of patients with incurable progressive disease and their families in South Africa and Uganda: multicentre qualitative study.* Bmj, 2009. **338**: p. b1326.
14. Selman, L.E., I.J. Higginson, G. Agupio, et al. *Quality of life among patients receiving palliative care in South Africa and Uganda: a multi-centred study.* Health Qual Life Out, 2011. **9**(1): p. 21.
15. Selman, L., R. Harding, I. Higginson, et al. *Spiritual wellbeing in sub-Saharan Africa: the meaning and prevalence of 'feeling at peace'.* BMJ Support Palliat Care, 2011. **1**(Suppl 1): p. A22.
16. Selman, L., R.J. Siegert, I.J. Higginson, et al. *The MVQOLI successfully captured quality of life in African palliative care: a factor analysis.* Journal of clinical epidemiology, 2011. **64**(8): p. 913-24.
17. Selman, L., R.J. Siegert, I.J. Higginson, et al. *The "Spirit 8" successfully captured spiritual well-being in African palliative care: factor and Rasch analysis.* Journal of clinical epidemiology, 2012. **65**(4): p. 434-43.
18. Streid, J., R. Harding, G. Agupio, et al. *Stressors and resources of caregivers of patients with incurable progressive illness in sub-Saharan Africa.* Qualitative health research, 2014. **24**(3): p. 317-28.

For peer review only - http://bmjopen.bmj.com/site/about/guidelines.xhtml

19. Bausewein, C., B.A. Daveson, D.C. Currow, et al. *EAPC White Paper on outcome measurement in palliative care: Improving practice, attaining outcomes and delivering quality services – Recommendations from the European Association for Palliative Care (EAPC) Task Force on Outcome Measurement.* Palliative Medicine, 2015. **30**(1): p. 6-22.
20. Harding, R., L. Selman, G. Agupio, et al. *Validation of a core outcome measure for palliative care in Africa: the APCA African Palliative Outcome Scale.* Health Qual Life Out, 2010. **8**: p. 10-10.
21. Blum, D., L.E. Selman, G. Agupio, et al. *Self-report measurement of pain & symptoms in palliative care patients: a comparison of verbal, visual and hand scoring methods in Sub-Saharan Africa.* Health Qual Life Outcomes, 2014. **12**(1): p. 118.
22. Harding, R., L. Selman, G. Agupio, et al. *Validation of a core outcome measure for palliative care in Africa: the APCA African Palliative Outcome Scale.* Health and Quality of Life Outcomes, 2010. **8**.
23. Harding, R., L. Selman, V.M. Simms, et al. *How to analyze palliative care outcome data for patients in Sub-Saharan Africa: an international, multicenter, factor analytic examination of the APCA African POS.* J Pain Symptom Manage, 2013. **45**(4): p. 746-52.
24. Beratarrechea, A., A.G. Lee, J.M. Willner, et al. *The Impact of Mobile Health Interventions on Chronic Disease Outcomes in Developing Countries: A Systematic Review.* Telemedicine and e-Health, 2013. **20**(1): p. 75-82.
25. Peiris, D., D. Praveen, C. Johnson, et al. *Use of mHealth Systems and Tools for Non-Communicable Diseases in Low- and Middle-Income Countries: a Systematic Review.* J. of Cardiovasc. Trans. Res., 2014: p. 1-15.
26. Betjeman, T.J., S.E. Soghoian, and M.P. Foran. *mHealth in sub-Saharan Africa.* Int. J. Telemedicine Appl., 2013. **2013**: p. 6-6.
27. World Health Organization, *Recommendations on digital interventions for health system strengthening*. 2019, World Health Organization: Geneva, Switzerland.
28. Allsop, M.J., E. Namisango, and R.A. Powell. *A survey of mobile phone use in the provision of palliative care services in the African region and priorities for future development.* Journal of Telemedicine and Telecare, 2018. **25**(4): p. 230-40.
29. Namisango, E., C. Ntege, E.B.K. Luyirika, et al. *Strengthening pharmaceutical systems for palliative care services in resource limited settings: piloting a mHealth application across a rural and urban setting in Uganda.* BMC Palliative Care, 2016. **15**(1): p. 20.
30. Allsop, M.J., R.A. Powell, and E. Namisango. *The state of mHealth development and use by palliative care services in sub-Saharan Africa: a systematic review of the literature.* BMJ Supportive & Palliative Care, 2016: p. bmjspcare-2015-001034.
31. Ajayi, I., O. Iken, R.A. Powell, et al. *Palliative care research in western Africa.* European Journal of Palliative Care, 2014. **21**(1): p. 45-47.
32. Merriman, A., *Audacity to Love: The Story of Hospice Africa*. 2010, Ireland: Irish Hospice Foundation.
33. Soyannwo, O. *Hospice and palliative care development in West Africa sub region: A review.* African Journal of Anaesthesia and Intensive Care, 2013. **13**: p. 10-13.
34. Omoyeni, N., O. Soyannwo, O. Aikomo, et al. *Home-based palliative care for adult cancer patients in Ibadan-a three year review.* Ecancermedicalscience, 2014. **8**: p. 490.
35. O'Brien, M., F. Mwangi-Powell, I.F. Adewole, et al. *Improving access to analgesic drugs for patients with cancer in sub-Saharan Africa.* The Lancet. Oncology, 2013. **14**(4): p. e176-82.
36. O'Brien, M., A. Schwartz, and L. Plattner. *Treat the Pain Program.* Journal of Pain and Symptom Management, 2018. **55**(2): p. S135-S39.
37. van Gurp, J., O. Soyannwo, K. Odebunmi, et al. *Telemedicine's Potential to Support Good Dying in Nigeria: A Qualitative Study.* PloS one, 2015. **10**(6): p. e0126820.
38. Rhee, J.Y., E. Luyirika, E. Namisango, et al., *APCA Atlas of Palliative Care in Africa*. 2017, Texas, USA: IAHPC Press.

For peer review only - http://bmjopen.bmj.com/site/about/guidelines.xhtml

39. The Economist Intelligence Unit, *The 2015 Quality of Death Index: Ranking palliative care across the world* 201, The Economist: London, UK.
40. Harding, R., V. Simms, S. Penfold, et al. *Availability of essential drugs for managing HIV-related pain and symptoms within 120 PEPFAR-funded health facilities in East Africa: a cross-sectional survey with onsite verification.* Palliat Med, 2014. **28**(4): p. 293-301.
41. Merriman, A. and R. Harding. *Pain control in the African context: the Ugandan introduction of affordable morphine to relieve suffering at the end of life.* Philosophy, ethics, and humanities in medicine : PEHM, 2010. **5**: p. 10-10.
42. Khumalo, T. and V. Maasdorp. *The Island Hospice model of palliative care.* Ecancermedicalscience, 2016. **10**: p. 654-54.
43. United Nations Children's Fund, *Assessment of the Need for Palliative Care for Children: Three Country Report: South Africa, Kenya and Zimbabwe*, S.R. Connor and C. Sisimayi, Editors. 2013.
44. Zimbabwe Ministry of Health and Child Care, *The National Palliative Care Policy*, The Department of Epidemiology & Disease Control Non-Communicable Diseases Unit, Zimbabwe Ministry of Health and Child Care: Harare, Zimbabwe. 2014.
45. African Palliative Care Association, *Review of the status of palliative care in ten Southern African countries.* 2016, African Palliative Care Association: Kampala, Uganda.
46. Worldwide Palliative Care Alliance, *Global Atlas of Palliative Care at the End of Life*, S.R. Connor and M. Bermedo, Editors. 2014, World Health Organization: London.
47. Gale, N.K., G. Heath, E. Cameron, et al. *Using the framework method for the analysis of qualitative data in multi-disciplinary health research.* BMC Medical Research Methodology, 2013. **13**(1): p. 117.

For peer review only

Appendix A: Palliative care reports undertaken in participating countries over the last 15 years

Country	Report	Key findings
Nigeria	Federal Ministry of Health: Treat the Pain (2012 – 2015):	• Around 177,000 people were estimated to have died in moderate or severe pain from HIV or cancer.<br>• The utilized narcotic medicines such as morphine was enough to treat only 266 people, representing 0.2% coverage of pain treatment
	Hospital Pain Free Initiative (2015 – date):	• Program to improve access to essential pain medicines to reduce needless suffering from cancer<br>• Pharmacist employed into Federal Ministry of Health specifically to coordinate the project<br>• 19.2kg of pulverized morphine imported, enough to treat about 3,000 patients.<br>• Strengthening the skills of health workers in pain assessment and management and equip them to provide high- quality pain treatment to their patients in line with World Health Organization (WHO) guidelines.<br>• Four teaching hospitals selected for pilot project (Based on their palliative care activity and utilization of oral morphine solution). Expanded to 15 by 2018
	National Comprehensive Cancer Network Harmonized Guidelines for Sub-Saharan Africa (2018 - 2019):	• Stakeholder workshops held leading to regional resources created as part of a collaborative efforts to combat rising cancer rates and unique care including palliative care
Uganda	Ministry of Health and National Drug clinical guidelines (2003):	• Nurses and Clinical officers are allowed to prescribe morphine for pain management
	Uganda Health Strategic plan, 2006–2011:	• Palliative Care is fully incorporated into the National Health Sector Strategic plan for the first time
	Evidence base for palliative care (2015)[48]	• A lack of policies and guidelines to support the development of palliative care was noted<br>• Plans to take forward palliative care development as part of the national health strategy
	Implementing a palliative care nurse leadership	• Trained palliative care nurses and clinical officers can effectively prescribe oral morphine

For peer review only

	fellowship program in Uganda (2016)[49]	
	Atlas of palliative care development (2017):	• Number of palliative care service outlets reported at 229<br>• Number of pediatric palliative care services reported at 2<br>• Has specialised training programmes for palliative care<br>• Palliative care services per million inhabitants reported at 5.87<br>• Has a Palliative Care National Association<br>• Oral morphine is produced locally
	National Cancer Control plan (2018):	• In the background based on an evidence brief for need for control strategy palliative care is recognised as part of the cancer control plan , and that it should be evidence based<br>• The Development of the NCCPs is underway, with a draft in place and palliative care is inclusive<br>• The NCCP addresses six key elements: (1) prevention, (2) early detection and screening, (3) diagnosis and treatment, (4) palliative care and (5) policy advocacy (6) research
	National eHealth strategy (2018):	• Baseline survey shows fragmented landscape of ICT pilot projects and numerous data and health information system (HIS) silos with significant barriers to the effective sharing of information between healthcare participants.<br>• Uganda adopts eHealth strategy to guide the use of ICT in supporting health sector transformation by addressing
Zimbabwe	WHO pilot study of palliative care in five African counties (2004): https://www.who.int/cancer/palliative/projectproposal/en/	• Estimated 1/60 people in Zimbabwe in need of palliative care<br>• Low level of palliative integration in the health system despite long history<br>• Training is a major strength<br>• Erratic pain relief
	African Palliative Care Association Policy and Gender Review in 10 countries (2010):	• Minimal coverage of palliative care<br>• No policies reviewed facilitated opioid availability<br>• Revealed gaps to be addressed to scale up integration of palliative care<br>• Recommended development of palliative care policy to guide actions and support for palliative care issues at national level

For peer review only

	National Palliative Care Situational Analysis commissioned by HOSPAZ (2012):	• Lack of knowledge and skills including listening and communication skills among care providers at all levels<br>• Need exists for palliative care training, bereavement counselling and home-based care<br>• Lack of knowledge / expertise in pain assessment and management using WHO analgesic ladder<br>• Unavailability and erratic supply of pain control medicines, especially strong opioids<br>• Children either not part of palliative care process or health providers have difficulty in handling their unique requirements (e.g. pain assessment and counselling)<br>• Poor integration of palliative care into public sector<br>• Lack of palliative care awareness and limited provision
	UNICEF and ICPCN Assessment of the Need for Palliative Care for Children: Three Country Report (2013):	• Number of children benefiting from palliative care services significantly lower than the estimated need (6% of specialised need)<br>• Lack of knowledge and adequate understanding of palliative care and children's palliative care among health professionals<br>• Lack of palliative care awareness by general public hence limited demand<br>• Poor pain management in children<br>• Morphine prescribing limited to medical doctors which is problematic
	African Palliative Care Association Morphine Survey (2015):	• Some progress toward integration of palliative care into health systems<br>• Advocacy for legal and policy changes recommended for nurse-prescribing of strong pain-relieving drugs, including opioids<br>• Opioid use in 2012 only 10.1kg instead of expected 177kg deduced from disease burden<br>• Recommended relevant knowledge on use of opioid analgesics to be integrated into medical and nursing school curricula
	Treat the Pain: A Country Snapshot (2016):	• Estimated 80% of cancer deaths (2012/2013) experience moderate to severe pain<br>• Average morphine consumption for 2011-2013 estimated 15.6kg, much lower than anticipated use<br>• Coverage of deaths in pain with treatment: 6%

For peer review only

Appendix B: Facilities involved in identification and recruitment of participants

**Country**	**Facility name**	**Type of facility**	**Participants to recruit from facility**
Nigeria	Lagos University Teaching Hospital	Tertiary care facility	Patients, caregivers, health professionals
	Sebeccly Cancer Care and Support Center	Private not-for- profit, community organisation	Patients, caregivers, health professionals
	National Ministry of Health, Nigeria	National government office	Policymakers
Uganda	Uganda Cancer institute (UCI)	Tertiary care medical facility	Patients, caregivers, health professionals
	Makerere Palliative Care Unit, Mulago Hospital	National referral hospital	Patients, caregivers, health professionals
	Kawempe Home Care	Private not-for- profit, community organisation	Patients, caregivers, health professionals
	Hospice Africa Uganda	Private not-for- profit, organisation with three facilities across Uganda	Patients, caregivers, health professionals
	Uganda Ministry of Health	National government office	Policymakers
Zimbabwe	Chitungwiza Hospital	Central hospital	Patients, caregivers, health professionals
	Parirenyatwa Group of Hospitals	Central hospital	Patients, caregivers, health professionals
	National Ministry of Health staff	National government office	Policymakers
	Island Hospice and Healthcare	Private not-for- profit, community organisation	Patients, caregivers, health professionals

For peer review only
